# LncRNA *HBL1* is required for genome-wide PRC2 occupancy and function in cardiogenesis from human pluripotent stem cells

**DOI:** 10.1242/dev.199628

**Published:** 2021-06-28

**Authors:** Juli Liu, Sheng Liu, Lei Han, Yi Sheng, Yucheng Zhang, Il-Man Kim, Jun Wan, Lei Yang

**Affiliations:** 1 Department of Pediatrics, Indiana University School of Medicine, Indianapolis, IN 46202, USA; 2 Department of Medical and Molecular Genetics, Indiana University School of Medicine, Indianapolis, IN 46202, USA; 3 Department of Obstetrics, Gynecology & Reproductive Sciences, University of Pittsburgh, Pittsburgh, PA 15213, USA; 4 Department of Anatomy, Cell Biology and Physiology, Indiana University School of Medicine, Indianapolis, IN 46202, USA; 5 Center for Computational Biology and Bioinformatics, Indiana University School of Medicine, Indianapolis, IN 46202, USA

**Keywords:** Long non-coding RNA, Polycomb repressive complex 2, Cardiac differentiation, Heart development, Pluripotent stem cells

## Abstract

Polycomb repressive complex 2 (PRC2) deposits H3K27me3 on chromatin to silence transcription. PRC2 broadly interacts with RNAs. Currently, the role of the RNA-PRC2 interaction in human cardiogenesis remains elusive. Here, we found that human-specific heart brake lncRNA 1 (*HBL1*) interacted with two PRC2 subunits, JARID2 and EED, in human pluripotent stem cells (hPSCs). Loss of JARID2, EED or *HBL1* significantly enhanced cardiac differentiation from hPSCs. *HBL1* depletion disrupted genome-wide PRC2 occupancy and H3K27me3 chromatin modification on essential cardiogenic genes, and broadly enhanced cardiogenic gene transcription in undifferentiated hPSCs and later-on differentiation. In addition, ChIP-seq revealed reduced EED occupancy on 62 overlapped cardiogenic genes in *HBL1*^−/−^ and *JARID2*^−/−^ hPSCs, indicating that the epigenetic state of cardiogenic genes was determined by *HBL1* and JARID2 at pluripotency stage. Furthermore, after cardiac development occurs, the cytosolic and nuclear fractions of *HBL1* could crosstalk via a conserved ‘microRNA-1-JARID2’ axis to modulate cardiogenic gene transcription. Overall, our findings delineate the indispensable role of *HBL1* in guiding PRC2 function during early human cardiogenesis, and expand the mechanistic scope of lncRNA(s) that cytosolic and nuclear portions of *HBL1* could coordinate to orchestrate human cardiogenesis.

## Abstract (precis)

**Summary:** This study reveals the indispensable role of the lncRNA *HBL1* in guiding PRC2 function during early human cardiogenesis, and uncovers the crosstalk of the cytosolic and nuclear regions of *HBL1* to orchestrate human cardiac development.

## INTRODUCTION

Over the past decades, studies of heart development have focused on conserved gene regulatory mechanisms, including cardiac transcription factors (Diogo et al., 2015; Laflamme and Murry, 2011), microRNAs (Small and Olson, 2011) and epigenetic regulators (Bruneau, 2013; Liu et al., 2020), which controlled steps of cardiogenesis in multiple species from *Drosophila* to mouse. However, compared with rodents, the human heart exhibits unique properties, including distinctive morphogenesis and electrophysiology. These species-specific features suggest the existence of a novel genetic regulatory program underlying human heart development. Recently, accumulating evidence has demonstrated that long non-coding RNA (lncRNA) plays vital roles in stem cell differentiation, organogenesis and disease, including cardiac development and pathophysiology (Fatica and Bozzoni, 2014; Klattenhoff et al., 2013; Ounzain et al., 2015; Quinn and Chang, 2016; Schonrock et al., 2012). In particular, the low interspecies conservation of lncRNAs indicates their species-specific functions. Diverse roles of lncRNAs in regulating gene transcription have been found, including genomic imprinting, chromatin modification, chromosome organization, mRNA decay and microRNA sponge (Ponting et al., 2009; Rinn and Chang, 2012). Importantly, many lncRNAs epigenetically regulate gene expression by interacting with chromatin modification complexes (Kotake et al., 2011; Kugel and Goodrich, 2012; Pandey et al., 2008; Spitale et al., 2011; Tsai et al., 2010; Yap et al., 2010). For example, lncRNAs *Xist*, *Kcnq1ot1*, *Meg3*, *HOXA1* and *Bvht* bind the polycomb repressive complex 2 (PRC2) (Kaneko et al., 2014; Khalil et al., 2009; Klattenhoff et al., 2013; Pandey et al., 2008; Sanli et al., 2018; Zhao et al., 2010). PRC2 is composed of several key subunits, including Ezh1/2, Suz12 and Eed. Through Ezh2-mediated H3K27me3 chromatin modification, PRC2 represses gene transcription. Eed can allosterically activate the methyltransferase activity of PRC2, which ensures the propagation of H3K27me3 on nucleosomes (Margueron et al., 2009). Jarid2, which is a member of the Jumonji protein family histone demethylases with inactive catalytic activity, facilitates PRC2 recruitment to the promoters of target genes (Li et al., 2010). PRC2 is required for stem cell differentiation, as well as heart development, maturation and disease (Ai et al., 2017; Cifuentes-Rojas et al., 2014; Conway et al., 2018; Gilsbach et al., 2014; He et al., 2012a,b; Morey et al., 2015a; Sanulli et al., 2015; Walker et al., 2010). However, PRC2 has been found to broadly interact with many nascent RNAs (Beltran et al., 2016; Davidovich et al., 2013; Zhao et al., 2010), leading to a debate on the biological importance of RNA-PRC2 interaction. Although a recent study revealed that the RNA-binding capacity of PRC2 was required for its chromatin occupancy in human pluripotent stem cells (hPSCs) and in turn for cardiac differentiation (Long et al., 2020), RNA(s) that guides PRC2 deposition on specific target genes in hPSCs to control cardiogenic gene transcription still remains elusive.

In this study, we found human-specific heart brake lncRNA 1 (*HBL1*) directly interacted with two PRC2 subunits, jumonji and AT-rich interaction domain containing 2 (JARID2) and embryonic ectoderm development (EED), to form a *HBL1*-PRC2 complex in the nucleus of undifferentiated hPSCs. *JARID2*^−/−^ and *EED*-knockdown hPSCs both phenocopied *HBL1*^−/−^ hPSCs, showing significantly increased cardiomyocyte (CM) differentiation when compared with wild-type (WT) hPSCs. In hPSCs, *HBL1* was required for global PRC2 occupancy and H3K27me3 chromatin modification on promoters of essential cardiogenic genes. Consequently, knockout of *HBL1* broadly promoted transcription of cardiac genes in hPSCs and during hPSC differentiation. The interaction between JARID2 and EED was not dependent on *HBL1*; however, knockout of *HBL1* and *JARID2* in hPSCs led to prominently reduced EED occupancy on 62 overlapped essential cardiac genes. Thus, in hPSCs, nuclear *HBL1* interacts with JARID2 and EED to guide specific PRC2 occupancy on essential cardiogenic genes to suppress transcription. In addition, we have previously reported that cytosolic HBL1 modulated cardiac development from hPSCs by counteracting microRNA-1 (Liu et al., 2017), and here we identified that *JARID2* mRNA was a new target of microRNA-1. Therefore, in hPSCs, cytosolic and nuclear *HBL1* cross-talked to fine-tune cardiogenic gene expression via a conserved ‘microRNA-1-JARID2’ axis. Overall, we uncovered an lncRNA *HBL1*-microRNA-1-PRC2 regulatory network controlling early cardiogenic gene expression in hPSCs, and supported the functional importance of lncRNA-PRC2 interaction in human cardiogenesis.

## RESULTS

### *HBL1* interacts with PRC2 subunits JARID2 and EED

**Fig. 1.**
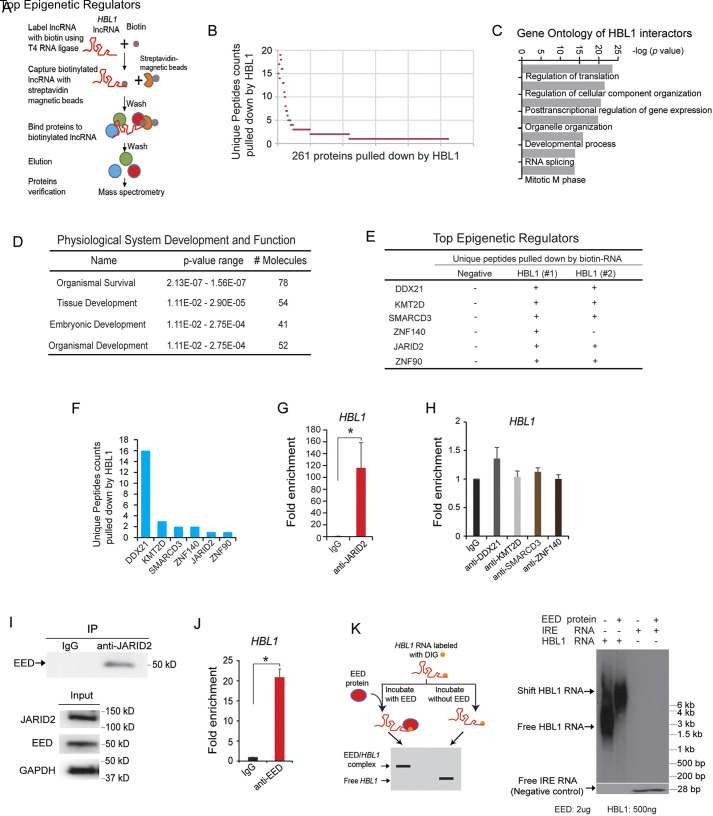
**LncRNA *HBL1* interacts with JARID2 and EED.** (A) Scheme for identification of *HBL1*-interacting proteins by using biotinylated *HBL1* and mass spectroscopy (MS). Pull-down experiments using biotin-*HBL1* were performed in two replicates. (B) All proteins pulled down by biotin-*HBL1*. Proteins were identified by unique peptides from MS. Pull-down experiment was performed with two replicates. (C) Gene Ontology analysis of all proteins pulled down by biotin-*HBL1*. (D) Physiological system development and function analysis for all protein candidates from MS data. (E) Top epigenetic regulators of all *HBL1* interactors based on the unique peptide counts identified by MS. (F) Top epigenetic regulators pulled down by biotin-*HBL1*. (G) Detection of interaction between *HBL1* and JARID2 by RIP-RT-qPCR. (H) Detection of interactions between *HBL1* and other proteins by RIP-RT-qPCR. (I) Co-IP-western blotting to detect interaction of JARID2 and EED proteins. (J) Interaction of *HBL1* and EED shown by RIP-RT-qPCR. (K) REMSA shows the interaction of *HBL1* with EED protein. *IRE* RNA was used as a negative control. All experiments were performed in triplicate. Data are mean±s.d. *n*=3, *P<0.05 (two-tailed unpaired Student's *t*-test). See also Fig. S1.

Previously, we have found lncRNA *HBL1* expression in both cytosol and nuclei of undifferentiated human induced pluripotent stem cells (hiPSCs), and cytosolic *HBL1* regulated CM differentiation from hiPSCs via counteracting microRNA-1 (Liu et al., 2017). To explore the role of *HBL1* in the nucleus, *HBL1* was biotin-labeled to pull down all interacting proteins in hiPSCs, followed with mass spectrometry (Fig. 1A). Through unique peptides identification, 261 proteins were found to interact with biotin-*HBL1* (Fig. 1B; Table S1). Both gene ontology (GO) and ingenuity pathway analysis (IPA) functional enrichment analyses were conducted, which found that *HBL1*-associating proteins were functionally related to gene expression and tissue development etc. (Fig. 1C,D; Fig. S1A). We then focused on nuclear epigenetic regulators and found that *HBL1* pulled down epigenetic regulators DDX21, SMARCD3 (BAF60C), ZNF140 and JARID2 (Fig. 1E,F). Next, RNA immunoprecipitation (RIP)-RT-qPCR was performed to verify interactions between *HBL1* and those nuclear protein factors. RIP-RT-qPCR data showed that anti-human JARID2 antibody enriched *HBL1* >100-fold more than IgG (Fig. 1G). However, antibodies against other factors did not prominently enrich *HBL1* when compared with IgG (Fig. 1H). Fig. S1B,C shows the expression of *HBL1* in input and no enrichment of control β-actin RNA by anti-human JARID2 antibody. These results demonstrate that RNA pull-down and RIP assays may generate discrepancies. However, data from both assays revealed that JARID2 was a nuclear epigenetic factor interacting with *HBL1*. JARID2 has been previously reported to bind PRC2 to regulate differentiation of mouse embryonic stem cells (ESCs) (Landeira et al., 2010; Pasini et al., 2010; Sanulli et al., 2015). Therefore, we next conducted protein co-immunoprecipitation (Co-IP) in undifferentiated hiPSCs and found that JARID2 antibody pulled down EED, which is a key component of PRC2 (Fig. 1I). Given that a single lncRNA could interact with multiple proteins (McHugh et al., 2015), we then asked whether *HBL1* could also interact with other PRC2 components, as well as JARID2. An lncRNA-protein interaction prediction was conducted using lncPro (Lu et al., 2013a) (Fig. S1D), which suggested *HBL1* might interact with JARID2 and EED, but not EZH2 or SUZ12. Therefore, we performed RIP-RT-qPCR and found that anti-EED antibody pulled down *HBL1*, but not the control β-actin RNA (Fig. 1J; Fig. S1B). Finally, an RNA electrophoretic mobility shift assay (REMSA) showed that mobility of *HBL1* RNA was retarded by adding EED protein (Fig. 1K). The shift of control *IRE* RNA was not affected by EED protein (Fig. 1K), but was retarded by adding liver extract as the positive control for REMSA (Fig. S1E). In addition, REMSA showed that *HBL1* could not interact with the other PRC2 complex members EZH2 and SUZ12 (Fig. S1F-H). Altogether, these results reveal that, in hiPSCs, *HBL1* interacts with two PRC2 subunits, JARID2 and EED.

### Knockdown of EED promotes cardiac differentiation from hPSCs

**Fig. 2.**
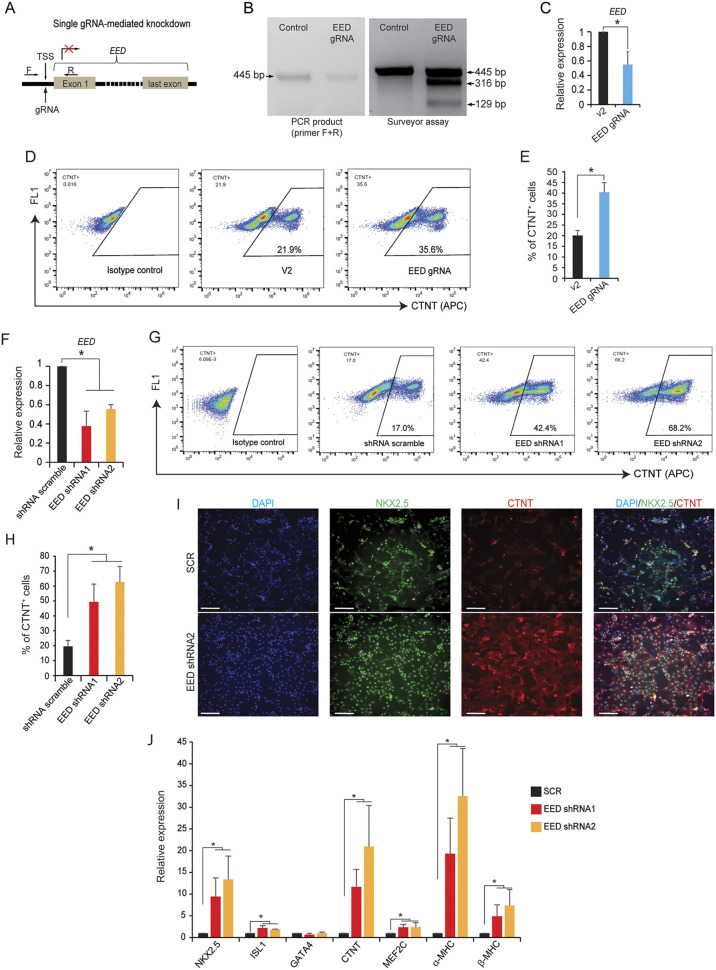
**Knockdown of *EED* promotes cardiac differentiation from hPSCs.** (A) Scheme for CRISPR/Cas9-mediated *EED*-knockdown in H9 hESCs using a single gRNA targeting the TSS. (B) Surveyor assay was used to detect genomic editing on human *EED* TSS. (C) Relative *EED* mRNA expression shown by RT-qPCR in H9 hESCs. (D,E) Cardiac differentiation from H9 hESCs was induced under a monolayer differentiation condition for 8 days, followed with FACS detection of percentage of CTNT^+^ CMs. (F) Generation of two *EED*-knockdown H9 hESC lines using shRNAs, followed by RT-qPCR detection of relative *EED* mRNA expression. (G,H) WT and *EED*-knockdown H9 hESCs were induced under monolayer cardiac differentiation condition for 8 days, followed by detection of percentage of CTNT^+^ CMs by FACS. (I) Immunostaining shows expression of two cardiomyocyte markers NKX2.5 and CTNT in SCR control and *EED* shRNA-knockdown H9 hESC-derived CMs. (J) RT-qPCR analysis of cardiac genes expressions in WT and *EED*-knockdown h9 hESCs after 8 days of cardiac differentiation. Data are mean±s.d. *n*=3, *P<0.05 [two-tailed unpaired Student's *t*-test (two groups) and a one-way ANOVA (multiple groups)]. Scale bars: 50 μm. See also Fig. S2.

Given the *HBL1*-EED interaction, we sought to study the role of EED in human cardiac differentiation from hPSCs. As *Eed*^−/−^ mouse (m)ESCs lost stemness (Morey et al., 2015b) and *EED*^−/−^ human (h)ESCs exhibited spontaneous differentiation (Shan et al., 2017), we chose to knock down *EED* in H9 hESCs and hiPSCs (Liu et al., 2017). We designed a single gRNA to target the transcriptional start site (TSS) of *EED*, which disrupted *EED* transcription (Fig. 2A). Surveyor assay detected genome editing on the TSS of *EED* (Fig. 2B), which decreased the *EED* mRNA level to ∼50% of that in WT H9 hESCs (Fig. 2C). After conducting cardiac differentiation for 8 days using a monolayer differentiation method (Fig. S2A), increased percentages of the cardiac muscle troponin T-positive (CTNT^+^) CMs were generated from the *EED* knockdown H9 hESCs compared with the empty vector control (Fig. 2D,E). Knockdown of *EED* in S3 hiPSCs also enhanced CM differentiation efficiency (Fig. S2B) and phenocopied *EED* knockdown in H9 hESCs. To further confirm the results from CRISPR/Cas-9-mediated *EED* knockdown, we next designed two shRNAs to knock down *EED* in H9 hESCs and hiPSCs (Fig. S2C). *EED*-shRNAs significantly decreased EED expression level (Fig. 2F) and increased ratios of CTNT^+^ CMs derived from H9 hESCs (Fig. 2G,H) and hiPSCs (Fig. S2D). Representative images from immunocytochemistry show increased NK2 homeobox 5-positive (NKX2.5^+^) and CTNT^+^ CMs derived from H9 hESCs after *EED* knockdown when compared with control (Fig. 2I). RT-qPCR analysis further revealed that knockdown of *EED* enhanced the expression of multiple cardiac genes after cardiac differentiation, including cardiogenic transcription factors (TFs) *NKX2.5* (*NKX2-5*), ISL LIM homeobox 1 (*ISL1*) and myocyte enhancer factor 2C (*MEF2C*), and sarcomere genes *CTNT* (*TNNT2*) and myosin heavy chain 6/7 (*MYH6*/*7*, encoding α/β-MHC) (Fig. 2J). These results demonstrate that loss of EED enhanced cardiac differentiation from hPSCs, which phenocopied loss of *HBL1* (Liu et al., 2017) and implies a functional interplay between *HBL1* and EED-PRC2.

### *HBL1* directs genome-wide PRC2 occupancy and H3K27me3 modification on promoters of essential cardiogenic genes

**Fig. 3.**
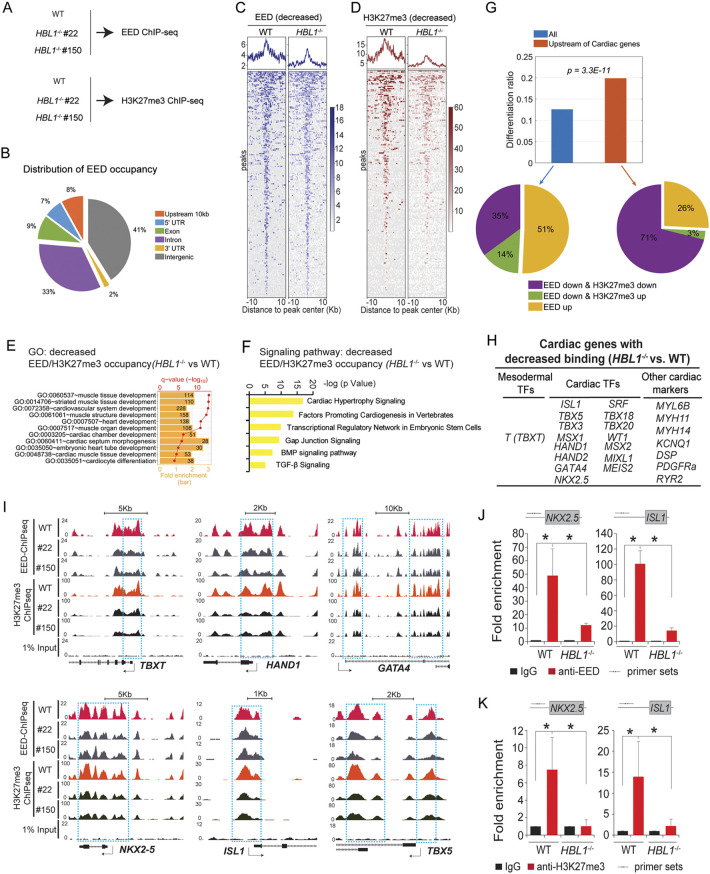
***HBL1* recruits EED occupancy on promoters of key mesodermal and cardiogenic genes.** (A) Schematic for ChIP-seq experiments on WT and *HBL1*^−/−^ hiPSCs using specific antibodies. (B) Distribution of EED occupancy on different genome locations. (C) Decreased EED binding signals around the EED peak centers (*HBL1*^−/−^ versus WT) (average for duplicates). (D) Decreased H3K27me3 modifications around the H3K27me3 peak centers (*HBL1*^−/−^ versus WT) (average for duplicates). (E) GO terms enriched in all genes with decreased EED/H3K27me3 binding activities (*HBL1*^−/−^ versus WT). The bars present the fold enrichments of GO terms, and the red line and dots show their statistical significances (−log_10_ *q*-value). The numbers of genes associated with GO functions are listed in corresponding bars. (F) Signaling pathways associated with genes with decreased EED/H3K27me3 binding (*HBL1*^−/−^ versus WT). (G) Comparison of the percentage of EED/H3K27me3 differentially binding regions (*HBL1*^−/−^ versus WT) on the whole genome (blue bar) and the upstream of cardiac genes (red bar). The *P*-value was evaluated using the hypergeometric model. The pie chart shows the relationship between EED and H3K27me3 signal changes in the differentiation regions, focusing on those specific to EED down upon *HBL1* ablation. (H) The list of key mesodermal and cardiogenic genes with decreased EED/H3K27me3 binding (*HBL1*^−/−^ versus WT). (I) Representative genome browser peak tracks of key mesodermal and cardiogenic genes with decreased EED/H3K27me3 signals (*HBL1*^−/−^ versus WT). WT, wild-type hiPSCs. #22 and #150 are two *HBL1*^−/−^ hiPSCs clones. 1% Input is the control. Arrow shows the gene transcription direction. (J,K) EED (J) and H3K27me3 (K) occupancies detected by ChIP-qPCR on cardiac gene promoters. Arrows show the primer sets. Data are mean±s.d. *n*=3, *P<0.05 (ChIP-qPCR data comparisons use two-tailed unpaired Student's *t*-test). See also Fig. S3.

To study whether *HBL1* affects genome-wide PRC2 occupancy and H3K27me3 chromatin modification, ChIP-seq was performed using antibodies against EED and H3K27me3 in WT and *HBL1*^−/−^ (#22 and #150) hiPSCs (Fig. 3A). We found ∼24% of the 125,759 EED binding sites located in the upstream (up to 10 kb from TSS), 5′ untranslated region (UTR), or exon of coding genes (Fig. 3B). Notably, significantly reduced EED and H3K27me3 signals were found on the genome (Fig. 3C,D), which is associated with genes over-represented in the biological process of heart/muscle-related development (Fig. 3E) and cardiogenesis signaling pathways (Fig. 3F). Indeed, 15,834 (12.6% of 125,759) of all EED/H3K27me3 binding sites showed significant signal changes after *HBL1* was ablated (Fig. 3G, upper panel, blue bar), of which 49% were reduced and the other 51% were increased (Fig. 3G, lower left panel). Of the 49% of EED/H3K27me3 binding sites with reduced signal, 35.0% exhibited reduced signals for both EED and H3K27me3 depositions (Fig. 3G, lower left panel). Interestingly, the ratio of altered EED/h3K27me3 occupancy on the proximity (mostly upstream) of promoters of cardiogenic genes was significantly higher than that of genome (19.9% compared with 12.6%, *P*=3.3E-11 based on hypergeometric model) (Fig. 3G, upper panel). The list of cardiac genes was obtained from an unbiased database, the Human Protein Atlas database (https://www.proteinatlas.org). Of the proximity (mostly upstream) of cardiogenic gene promoters with altered EED/H3K27me3 deposition, 71% showed both reduced signals of H3K27me3 and EED occupancy (Fig. 3G, lower right). These results indicated that loss of *HBL1* specifically reduced PRC2 occupancy and PRC2-deposited H3K27me3 modification on cardiac gene promoters, including key mesodermal formation factor *T* (*TBXT*), key cardiogenic TFs such as *HAND1*/*2*, *GATA4*, *NKX2.5*, *ISL1*, *TBX3*/*5*/*18*/*2*, and cardiac functional genes such as *KCNQ1*, *RYR2*, etc. (Fig. 3H; Table S2). In *HBL1*^−/−^ hiPSCs, decreased EED occupancy and H3K27me3 modification were observed on the promoter regions of essential cardiogenic genes as shown by the genome browser tracks (Fig. 3I). However, we did not observe significant changes of EED and H3K27me3 occupancies on promoters of other lineage genes (Fig. S3). Finally, ChIP-qPCR validated the ChIP-seq results (Fig. 3J,K), showing significantly decreased EED occupancy and H3K27me3 deposition on the promoter regions of two essential cardiogenic TFs, *NKX2.5* and *ISL1*, in *HBL1*^−/−^ hiPSCs compared with WT hiPSCs. Together, these data demonstrate that, in undifferentiated hPSCs, recruitment of functional PRC2 on promoters of essential cardiogenic genes is dependent on *HBL1*.

### *HBL1* depletion promotes transcription of cardiogenic genes

**Fig. 4.**
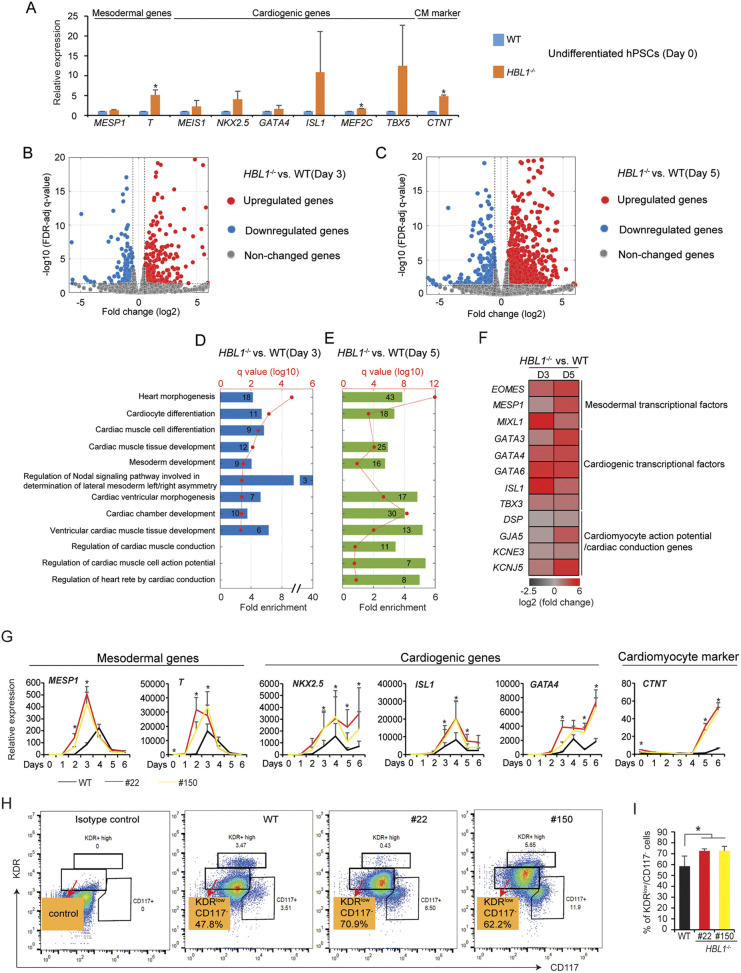
***HBL1* depletion promotes mesodermal and cardiogenic gene transcription.** (A) RT-qPCR analysis of relative mRNA expression of mesodermal and cardiac genes in undifferentiated hiPSCs (D0). (B,C) Volcano plots show all genes with differential expression levels (*HBL1*^−/−^ versus WT) on D3 (B) and D5 (C) of cardiac differentiation from hiPSCs. (D,E) GO functional enrichment analysis of all upregulated genes (*HBL1*^−/−^ versus WT) on D3 (D) and D5 (E) of cardiac differentiation from hiPSCs. GO is analyzed using DAVID. (F) Heatmap of key mesodermal TFs, cardiogenic TFs and cardiac functional gene expression profiles (*HBL1*^−/−^ versus WT) on D3 and D5 of cardiac differentiation from hiPSCs. (G) RT-qPCR analysis of relative mRNA expression of mesodermal genes, cardiogenic genes and cardiomyocyte marker in WT and *HBL1*^−/−^ hiPSCs during cardiac differentiation. RNAs were collected every day from D0 to D6. (H,I) Percentages of multipotential cardiac progenitors (KDR^low^/CD117^−^ population) were measured by FACS after cardiac differentiation for 6 days under EB differentiation conditions. Data are mean±s.d. *n*=3, *P<0.05 (two-tailed unpaired Student's *t*-test). See also Fig. S4.

Although loss of *HBL1* in hiPSCs reduced PRC2 occupancy and H3K27me3 deposition on cardiogenic gene promotors, RT-qPCR analysis also detected altered expression levels of key cardiac genes in *HBL1*^−/−^ hiPSCs (Fig. 4A). The expression levels of *MESP1*, *T*, *MEIS1*, *NKX2.5*, *GATA4*, *ISL1*, *MEF2C*, *TBX5* and *CTNT* were all increased in undifferentiated *HBL1*^−/−^ hiPSCs compared with WT hiPSCs (Fig. 4A), suggesting that *HBL1* repression is required for initiating the earliest cardiogenic gene expression program. To further explore the global gene expression changes during cardiac differentiation, we collected RNAs at day (D) 3 and D5 from both WT and *HBL1*^−/−^ hiPSCs, followed with RNA-seq (Fig. S4A,B). The upregulated genes (*HBL1*^−/−^ versus WT) at D3 (Fig. 4B; Table S3) were enriched into events including heart morphogenesis, mesoderm development and nodal signaling pathways (Fig. 4D), suggesting that depletion of *HBL1* might enhance early cardiac mesodermal differentiation and commitment. At D5, the upregulated genes (Fig. 4C; Table S3) were enriched into events including cardiac chamber development and cardiac muscle action potential (Fig. 4E; Fig. S4C), suggesting that *HBL1* deficiency could also promote expression of genes crucial for later-stage cardiac muscle formation. The enhanced expression of representative marker genes responsible for mesoderm formation, cardiogenesis and CM function in *HBL*^−/−^ versus WT hiPSCs during cardiac differentiation are shown in Fig. 4F. Together, these data reveal that *HBL1* depletion triggers the transcriptional program for cardiogenesis from the PSC stage, and enhances global transcription of cardiogenic genes from the very early stage of the cardiac development process.

To further confirm that *HBL1* deficiency causes dynamic changes of key cardiogenic genes, we conducted RT-qPCR from D0 to D6 of cardiac differentiation to assess expressions of crucial mesodermal genes *MESP1* and *T*, early cardiogenic TFs *NKX2.5*, *ISL1* and *GATA4*, and cardiomyocyte sarcomere gene *CTNT* (Fig. 4G). Compared with WT cells, loss of *HBL1* significantly increased the expression level of these genes during cardiac differentiation, which also suggests the enhanced formation of cardiac progenitors from *HBL*^−/−^ hiPSCs. Therefore, we next compared differentiation efficiencies of multipotential cardiovascular progenitors (MCPs) (Yang et al., 2008) from WT and *HBL1*^−/−^ hiPSCs. Knockout of *HBL1* in hiPSCs (*HBL1*^−/−^ clones #22 and #150) increased differentiation efficiencies of multipotential cardiac progenitors (MCPs) (KDR^low^/CD117^−^ population) (Yang et al., 2008) at D6 (Fig. 4H,I) and CMs at D20 of differentiation (Fig. S4D,E) when compared with WT hiPSCs. These data demonstrate that *HBL1* plays a crucial role in controlling cardiogenic transcriptional programs during the early stage of human cardiogenesis.

### JARID2 depletion increases cardiac differentiation from hPSCs

**Fig. 5.**
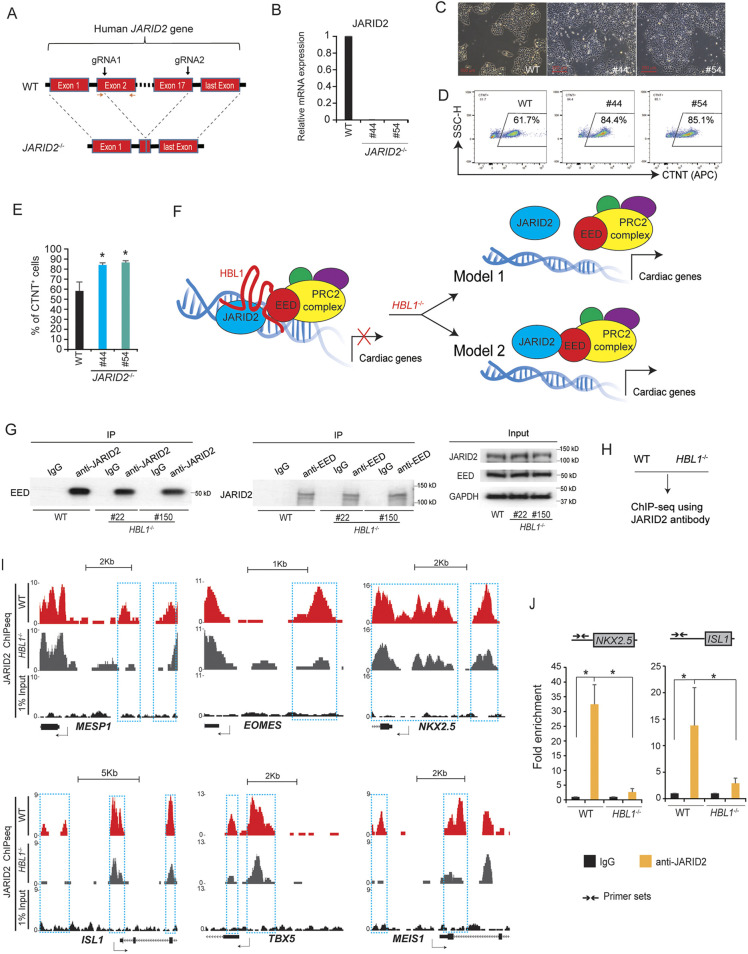
**JARID2 interacts with EED and *HBL1*.** (A) Scheme for generation of *JARID2*^−/−^ H9 hESCs using dual gRNAs and CRISPR/Cas-9. (B) Detection of relative *JARID2* mRNA expression in WT and *JARID2*^−/−^ hESCs using RT-qPCR. (C) Representative images of WT and *JARID2*^−/−^ hESCs cultured in mTesR1 medium. (D) Detection of percentages of CTNT^+^ CMs derived from WT and *JARID2*^−/−^ hESCs using flow cytometry. (E) Quantification of percentage of CTNT^+^ CMs derived from WT and *JARID2*^−/−^ hESCs. (F) Two proposed models showing *HBL1* interaction with the JARID2 and EED-PRC2 complexes (see text for explanation). (G) Co-IP western blotting to detect the interaction between JARID2 and EED. Left: pull-down of EED protein in WT and *HBL1*^−/−^ (#22, #150) hiPSCs using a specific JARID2 antibody by Co-IP. Right: pull-down of JARID2 protein in WT and *HBL1*^−/−^ (#22, #150) hiPSCs using a specific EED antibody by Co-IP. 1% Input is the Co-IP control. (H) ChIP-seq was performed on WT and *HBL1*^−/−^ hiPSCs using specific anti-JARID2 antibody. (I) Representative genome browser peak tracks of key mesodermal and cardiogenic genes with decreased JARID2 binding (*HBL1*^−/−^ versus WT hiPSCs). 1% Input is the control. Arrow shows the gene transcription direction. (J) ChIP-qPCR detects JARID2 occupancy on cardiac gene promoters in WT and *HBL1*^−/−^ hiPSCs. Arrows show the primer sets. Data are mean±s.d. *n*=3, *P<0.05 [two-tailed unpaired Student's *t*-test (two groups)]. See also Fig. S5.

*HBL1* interacts with both EED and JARID2 (Fig. 1). As function of JARID2 in human cardiogenesis was still unclear, we next investigated the role of JARID2 in cardiac differentiation from hPSCs. As shown in Fig. 5A, *JARID2* was knocked out in H9 hESCs using CRISPR/Cas-9 with two gRNAs targeting exon 2 and exon 17 of *JARID2*. From a total of 192 H9 hESC clones, nine *JARID2*^−/−^ clones were identified with a 4.2% knockout (KO) efficiency (Fig. S5A,B). Expression of *JARID2* was undetectable in *JARID2*^−/−^ H9 hESCs (Fig. 5B; Fig. S5C). *JARID2*^−/−^ hESC clones exhibited normal morphology without visible spontaneous differentiation (Fig. 5C; Fig. S5D). The expression level of pluripotency marker gene *OCT4* (*POU5F1*) in *JARID2*^−/−^ hESCs was comparable with that in WT hESCs (Fig. S5E), indicating that JARID2 is dispensable for pluripotency. Next, WT and *JARID2*^−/−^ hESCs were differentiated into CMs. Significantly increased percentages of CTNT^+^ CMs were derived from *JARID2*^−/−^ than control hESCs (Fig. 5D,E), which phenocopied *HBL1*^−/−^ (Liu et al., 2017) and *EED*-knockdown hPSCs (Fig. 2E). These results demonstrate that *JARID2*, *HBL1* and *EED* all suppress cardiac differentiation from hPSCs.

### JARID2-EED interaction is not *HBL1* dependent

As JARID2 interacts with both EED and *HBL1*, we next asked whether the interaction between JARID2 and EED proteins was *HBL1*-dependent. We hypothesized two potential scenarios (Fig. 5F): (1) *HBL1* anchors JARID2 with the EED-PRC2 complex, and therefore loss of *HBL1* dissociates JARID2 from the EED-PRC2 complex (Model 1); (2) Loss of *HBL1* does not affect the JARID2-EED interaction (Model 2). Using Co-IP, we found that both anti-human JARID2 and EED antibodies could pull down EED and JARID2, respectively, from WT and *HBL1*^−/−^ hiPSCs (Fig. 5G), indicating that Model 2 was correct. Given that loss of *HBL1* did not dissociate the JARID2-EED interaction but reduced EED occupancy on promoters of essential cardiogenic genes (Fig. 3I), we then asked whether *HBL1* could also affect JARID2 occupancy on cardiogenic genes. ChIP-seq was performed using anti-human JARID2 antibody in *HBL1*^−/−^ and WT hiPSCs (Fig. 5H). In *HBL1*^−/−^ hiPSCs, reduced JARID2 occupancy was observed on promoter regions of crucial mesodermal genes *MESP1*, *EOMES* and *MEIS1*, and cardiogenic TFs including *NKX2.5*, *ISL1* and *TBX5* (Fig. 5I), which also exhibited reduced EED occupancy and H3K27me3 modification (Fig. 3I). Furthermore, using the same sets of primers from Fig. 3J, in *HBL1*^−/−^ versus WT hiPSCs, ChIP-PCR validated reduced JARID2 occupancy on the same promoter regions of *NKX2.5* and *ISL1* genes (Fig. 5J). Therefore, our data demonstrate that in hPSCs, the interaction of *HBL1* with JARID2 is crucial for PRC2 occupancy on cardiogenic genes.

### *HBL1* interacts with JARID2 to guide PRC2 occupancy on target genes in hPSCs

**Fig. 6.**
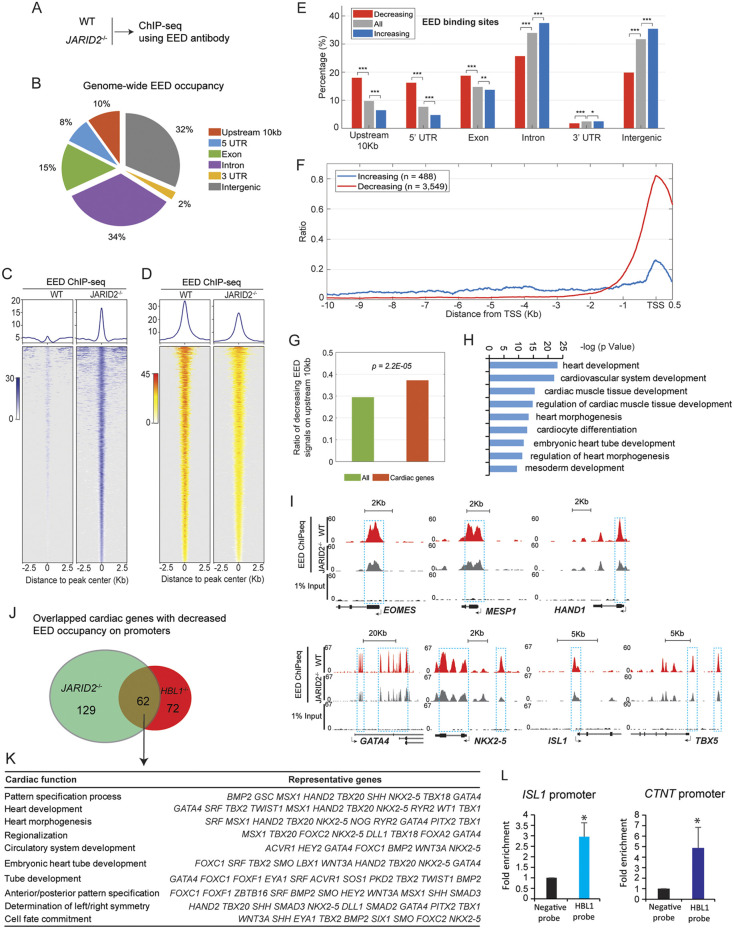
**EED occupancy on the promoters of key mesodermal and cardiogenic genes are regulated by JARID2.** (A) ChIP-seq was performed on WT and *JARID2*^−/−^ hESCs using specific anti-EED antibody. (B) Distribution of ChIP-seq peaks with EED binding relative to genomic elements. (C) Enrichment heatmap of ChIP-seq peaks with increased EED binding by JARID2 depletion. (D) Enrichment heatmap of ChIP-seq peaks with decreased EED binding by JARID2 depletion. (E) Comparison of all EED ChIP-seq peaks with decreased (red bar) or increased (blue bar) EED binding (*JARID2*^−/−^ versus WT) on different genomic locations. Gray bar means all peaks enriched by EED. *P<0.05, **P<0.01, ***P<0.001 (hypergeometric distribution). (F) Comparisons of ChIP-seq peaks with decreased (red line) or increased (blue line) EED binding (*JARID2*^−/−^ versus WT) on genomic locations close to the TSS. (G) Comparison of ratios of genes with decreased EED occupancy. Green bar represents all genes with decreased EED binding. Red bar represents cardiac genes with decreased EED binding. Hypergeometric distribution calculates the *P*-value. (H) GO analysis of all genes with decreased EED binding (*JARID2*^−/−^ versus WT). (I) Representative genome browser peak tracks of key mesodermal and cardiogenic genes with decreased EED binding (*JARID2*^−/−^ versus WT) in hESCs. WT, wild type hESCs. (J) Overlapped cardiac genes with decreased binding on promoters by EED in *JARID2*^−/−^ and *HBL1*^−/−^ hPSCs. (K) Functional enrichment analysis of overlapped 62 genes from J. (L) ChIRP results of HBL1 interaction with ISL1 and NKX2.5 promoters. Data are mean±s.d. *n*=3, *P<0.05 [two tailed unpaired Student’s *t*-test (two groups)]. See also Fig. S6.

To further study the impacts of *HBL1* and JARID2 on genome-wide PRC2 occupancy, we performed ChIP-seq using an anti-EED antibody in *JARID2*^−/−^ and WT hESCs (Fig. 6A; Fig. S6A). Of all EED-occupied genomic regions, 33% were located in regions that were 10 kb upstream, 5′ UTRs or within exons of coding genes (Fig. 6B). Knockout of *JARID2* altered genome-wide EED occupancy, with both increased (Fig. 6C) and decreased (Fig. 6D) signals. Interestingly, decreased EED occupancy was localized in genomic regions that are 10 kb upstream of TSSs, 5′ UTRs and within exons (Fig. 6E, red bars), whereas increased EED occupancy accumulated in other genomic regions including introns, 3′ UTRs and intergenic regions (Fig. 6E, blue bars; Fig. S6B). In addition, in *JARID2*^−/−^ hESCs, most decreased EED binding signals were found within 1 kb of the TSS (Fig. 6F), which are promoter regions. Moreover, when compared with all genes, significantly more cardiac genes exhibited reduced EED occupancy on promoter regions (*P*=2.2E-05, Fig. 6G), indicating that JARID2 selectively guided EED occupancy on promoters of cardiogenic genes. Indeed, GO analysis demonstrated that genes with decreased EED occupancy (*JARID2*^−/−^ versus WT hESCs, Table S4) were enriched into embryonic development events including heart and cardiovascular system development (Fig. 6H). Those genes included mesoderm formation genes *EOMES* and *MESP1*, and cardiogenic TFs such as *HAND1*, *GATA4*, *NKX2.5*, *ISL1* and *TBX5* (Fig. 6I). Next, we compared genes with reduced EED occupancy in *JARID2*^−/−^ and *HBL1*^−/−^ hPSCs. Reduced EED occupancy in both *JARID2*^−/−^ and *HBL1*^−/−^ hESCs was found in 834 genes (15.6% of all genes; Fig. S6C), and 62 out of 263 cardiac genes (23.6%, *P=1.5E-04*) (Fig. 6J; Fig. S6C). Importantly, the 62 overlapping cardiac genes are essential for heart development and morphogenesis (Fig. 6K). Lastly, Chromatin Isolation by RNA Purification (ChIRP) was conducted using biotin-probes targeting *HBL1*. We found that *HBL1* could bind promoter regions of *ISL1* and *CTNT* (Fig. 6L). Taken together, our data demonstrate that PRC2 occupancy on promoters of key cardiogenic genes is dependent on both JARID2 and *HBL1*, and the HBL1-JARID2-PRC2 complex determines the epigenetic state of essential cardiogenic genes in undifferentiated hPSCs.

### Cytosolic and nuclear *HBL1* cross talk via the ‘microRNA-1-JARID2’ axis

**Fig. 7.**
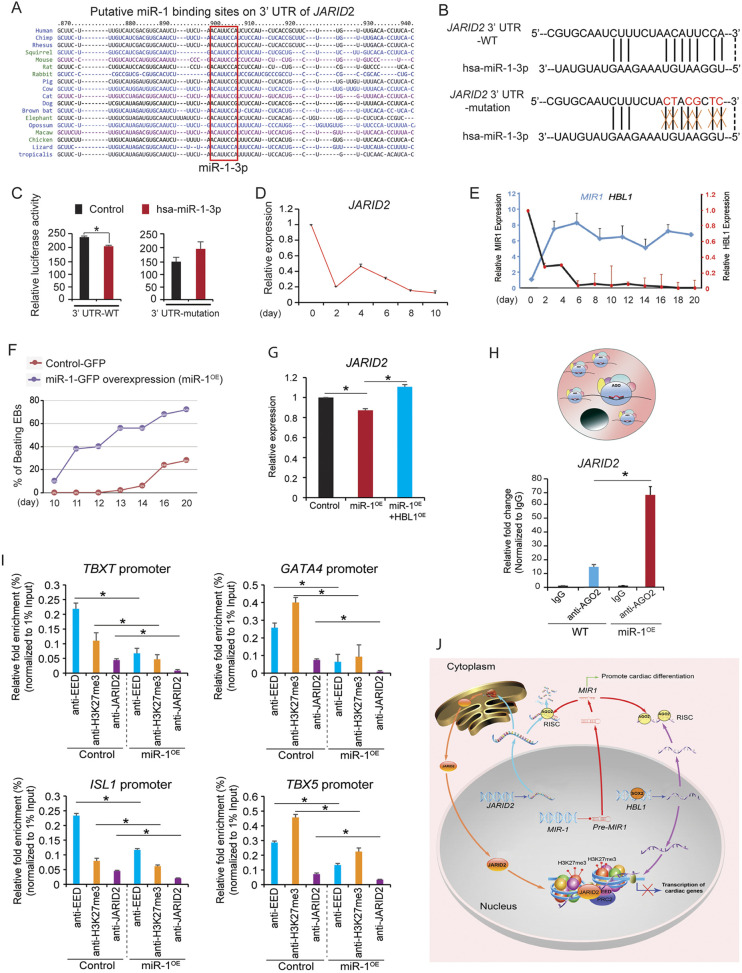
**JARID2 is a conserved target of microRNA-1.** (A) Putative miR-1 binding site on the 3′ UTR of *JARID2* gene across multiple species. (B,C) *JARID2* 3′ UTR containing WT and mutated putative hsa-miR-1 binding sites were cloned into pmiR-GLO vector, separately (B). Luciferase activities were detected in 293T cells co-transfected with WT or mutated JARID2 3′ UTR with or without hsa-miR-1-3p mimics. (C). (D) RT-qPCR detection of JARID2 expression dynamic during cardiac differentiation from hESCs. (E) Relative expression dynamics of hsa-miR-1 and *HBL1* during cardiac differentiation of hiPSCs. (F) Percentage of beating EBs from control and miR-1-overexpressing hiPSCs during cardiac differentiation. (G) Relative *JARID2* mRNA expression in hiPSCs detected by RT-qPCR. (H) Detection of hsa-miR-1 and JARID2 enrichments by anti-AGO2 antibody using RIP-RT-qPCR in hiPSCs. (I) ChIP-qPCR results show the occupancies of EED, H3K27me3 and JARID2 on promoters of crucial cardiogenic transcription factors in control and miR-1^OE^ hiPSCs. (J) Working model of *HBL1*, miR-1, JARID2 and EED-PRC2 regulatory network during human cardiogenesis. Data are mean±s.d. *n*=3. *P<0.05 [two-tailed unpaired Student's *t*-test (two groups)]. See also Fig. S7.

Previously, we reported that cytosolic *HBL1* regulated cardiomyocyte development from hPSCs by counteracting microRNA-1 (miR-1) (Liu et al., 2017). We then asked whether the cytosolic *HBL1*-miR-1 interaction could cross-talk with the nuclear *HBL1*-JARID2/EED-PRC2 complex. Although miR-1 was found to play an important role in cardiac differentiation from mESCs and hESCs (Lu et al., 2013b; Zhao et al., 2005), the downstream mechanism remains elusive. Using TargetScan (Supplementary Materials and Methods), we explored putative miR-1 targets among known nuclear epigenetic genes. Interestingly, *JARID2* was predicted as a top putative target of miR-1 (Fig. S7A), and the specific miR-1 binding site on the 3′ UTR of *JARID2* was highly conserved from *Xenopus* to human (Fig. 7A; Fig. S7B, supplementary Materials and Methods). Next, the WT *JARID2* 3′ UTR and the *JARID2* 3′ UTR containing a mutated miR-1 (MT) binding site were cloned into a pmiR-GLO luciferase reporter vector (Fig. 7B). The dual luciferase assay found that hsa-miR-1-3p repressed luciferase activity of WT, but not of MT 3′ UTR-*JARID2* (Fig. 7C), indicating that miR-1 bound *JARID2* in a sequence-specific manner. RT-qPCR detected the gradually decreased expression of *JARID2*, whereas the increased expression dynamic of miR-1, during cardiac differentiation from hPSCs (Fig. 7D,E), suggested that the dose of miR-1 could affect the *JARID2* expression level. As miR-1 was not expressed in hPSCs, we compared *JARID2* expression level in WT versus miR-1 overexpressing (miR-1^OE^) hiPSCs (Lu et al., 2013b). MiR-1 overexpression significantly promoted cardiomyocyte differentiation from hiPSCs with increased percentages of beating embryoid bodies (EBs) (Fig. 7F). MiR-1 overexpression also downregulated *JARID2* expression (Fig. 7G), which could be rescued by *HBL1* overexpression in miR-1^OE^ hiPSCs (Fig. 7G). As AGO2 protein, microRNA and its target RNAs form an RNA-induced silencing complex (RISC) (Chendrimada et al., 2005; Sontheimer, 2005), we performed RIP using an anti-AGO2 antibody in WT and miR-1^OE^ hiPSCs. *JARID2* mRNA was significantly enriched in the AGO2 complex of miR-1^OE^ hiPSCs compared with WT hiPSCs (Fig. 7H; Fig. S7C), showing that *JARID2* was a direct target of miR-1. Finally, we asked whether miR-1 overexpression could affect JARID2-PRC2 occupancy on cardiogenic genes by performing ChIP-qPCR. We found that miR-1 overexpression significantly reduced EED and JARID2 occupancy, as well as H3K27me3 disposition on promoters of crucial cardiogenic transcription factors (Fig. 7I). Altogether, these results demonstrate that cytosolic *HBL1*-miR-1 interaction could fine-tune *JARID2* expression levels after cardiac development occurs and miR-1 starts to express, which could subsequently affect nuclear JARID2/EED-PRC2 occupancy on cardiogenic genes. Therefore, both cytosolic and nuclear *HBL1* coordinate to precisely control cardiogenic gene transcription via the ‘microRNA-1-JARID2’ axis during early cardiac development from hPSCs (Fig. 7J).

## DISCUSSION

In this study, we found that nuclear *HBL1*-PRC2 interaction plays a crucial role in determining the chromatin modification status of cardiogenic genes in undifferentiated hPSCs. *HBL1* is required for recruiting PRC2 occupancy on key cardiogenic genes, which deposits H3K27me3 chromatin modification to suppress cardiogenic gene transcription. Importantly, once cardiac development from hPSCs initiates, the cytosolic *HBL1*-miR-1 and nuclear *HBL1*-JARID2/PRC2 mechanisms crosstalk to fine-tune cardiogenic gene transcription via a conserved microRNA-1-JARID2 axis. These findings uncover a complicated gene regulatory network by which lncRNA interacts with microRNA and epigenetic complex to govern cardiogenesis from hPSCs.

Several previous genome-wide studies reported the promiscuous interactions of PRC2 with many nascent RNAs (Beltran et al., 2016; Davidovich et al., 2013; Zhao et al., 2010), leading to an open question on whether lncRNA-PRC2 interaction could possess a specific biological function. Recently, Long and colleagues investigated the biological importance of PRC2-RNA interaction in hiPSCs by generating RNA-binding-defective PRC2, which did not bind RNAs and disrupted genome-wide PRC2 chromatin occupancy in hiPSCs (Long et al., 2020). Although this study concluded that PRC2 required RNA binding capacity for targeting chromatin in hPSCs, and in turn for defining CM differentiation, it did not address which RNA(s) could interact with PRC2 to regulate CM differentiation from hiPSCs. Zhao et al. (2008) reported that a short lncRNA, RepA RNA, could recruit the PRC2 complex to mediate X-chromosome inactivation, and Ezh2 served as a RepA binding subunit. RepA depletion abolished full-length Xist induction and H3K27me3 modification on the X-chromosome. Similarly, although using different models, our study proved the crucial role of the *HBL1*-PRC2 interaction in regulating epigenetic state and transcriptional activities of cardiogenic genes in hPSCs. When comparing our data with Long's study (Long et al., 2020), we also found similar results that both loss of *HBL1* and disruption of RNA binding on PRC2 reduced genome-wide PRC2 occupancy and H3K27me3 modification in undifferentiated hiPSCs, particularly on NKX2.5, a crucial cardiogenic TF. Theoretically, reduced PRC2 occupancy and H3K27me3 deposition should activate gene transcription (Blackledge et al., 2015; Cao et al., 2002; Schwartz and Pirrotta, 2013). However, Long's study reported that mutated PRC2 reduced NKX2.5 expression, as well as later CM differentiation, from hiPSCs (Long et al., 2020). It was contradictory to the canonical function of PRC2 in silencing gene transcription. Although further clarifications are required in that study, our studies have observed that loss of HBL1 could reduce PRC2 occupancy on key cardiac genes and consequently increase their transcription.

Some RNAs were reported to interact with the PRC2 complex. For example, Kanhere et al. (2010) identified a class of short RNAs, ∼50-200 nucleotides in length, which are transcribed from repressed polycomb target genes. Although they are not lncRNAs, they can interact with SUZ12, EED and EZH2 (Kanhere et al., 2010). Long et al. (2017) identified that EZH2 and EED are RNA-binding subunits. Zhang et al. (2019) also found that PRC2 complex core subunits, including EED, EZH2, SUZ12 and RBBP, have RNA-binding regions (RBR). Moreover, a recent study has shown that EZH2 and SUZ12 can interact with nascent RNAs (Rosenberg et al., 2021). In this study, our findings showed that lncRNA *HBL1* could interact with EED, but not EZH2 and SUZ12. These findings show that different subunits of the PRC2 complex can interact with different RNAs (long RNAs or short RNAs). We do not know why only EED could selectively interact with lncRNA *HBL1* in our study. One possibility is that different proteins have different binding motifs, some of which could be non-conserved or non-canonical RNA-binding motifs showing the non-canonical RNA-protein interaction (Long et al., 2017; Rosenberg et al., 2021; Zhang et al., 2019). More RNA-binding motifs should be investigated in future to understand the RNA-protein interactions.

The results from biotinylated *HBL1* pull-down (Fig. 1E) and the RIP (Fig. 1F) assays generated discrepancies, showing that, except for JARID2, protein factors pulled down by *HBL1* did not generate significant *HBL1* enrichment by RIP using antibodies against the same proteins. In the *HBL1* pull-down assay, biotinylated *HBL1* had to compete with endogenous *HBL1* to bind with interactive proteins. The affiliation of the endogenous proteins-*HBL1* interaction could determine the efficacies of using biotinylated-*HBL1* to pull down those proteins. Theoretically, proteins of higher affiliation with endogenous *HBL1* have a lower tendency to be pulled down by biotinylated-*HBL1*. In the RIP experiment, antibody was added to capture an endogenous protein that interacts with *HBL1*, and a protein with a higher affiliation with endogenous *HBL1* could be enriched with greater efficiency. Nevertheless, the technical discrepancies could be resolved by conducting both assays to investigate the real *HBL1*-binding protein(s); here, our data prove that JARID2 binds with *HBL1*.

Many lncRNAs, such as *HBL1*, are expressed in both the cytoplasm and nucleus. Whether and how cytosolic and nuclear fractions of an lncRNA could functionally interact remains elusive. Our data show that the cytosolic *HBL1*-miR-1 interaction (Liu et al., 2017) could indirectly affect occupancy of the nuclear *HBL1*-JARID2/PRC2 complex on target genes via modulating JARID2 expression. We found that miR-1 could target the 3′ UTR of *JARID2* mRNA to reduce its expression level, and JARID2 deficiency reduced PRC2 occupancy on cardiogenic genes. Notably, the miR-1 binding site on the 3′ UTR of *JARID2* is highly conserved across multiple species from *Xenopus* to human. Thus, this miR-1-JARID2 axis could be recognized as an evolutionarily important axis controlling cardiogenesis. This axis allows fine-tuning of nuclear PRC2 occupancy on cardiogenic genes through modulating miR-1 bioactivity in cytosol. Hence, cytosolic *HBL1* counteracts miR-1 to fine-tune the bioactivity of miR-1, which in turn determines the expression level of JARID2. After JARID2 protein enters the nucleus, both JARID2 and nuclear *HBL1* could co-determine the PRC2 occupancy on cardiogenic genes. Thus, this ensures the chromatin state of essential cardiogenic genes is under precise control by both cytosolic and nuclear mechanisms. It is also crucial for the sequential activation of a series of later-stage cardiac genes for cardiac fate commitment. Therefore, non-coding RNAs, including *HBL1* and miR-1, and PRC2 function together to fine-tune early human heart development from PSCs.

Our findings support a conclusion that the *HBL1*-PRC2 interaction facilitates the identification of specific PRC2 target genes, confirming a functional interplay between lncRNA and chromatin modification. In particular, our study demonstrates that the *HBL1*-PRC2 interaction plays a crucial role in initiating human cardiogenesis via globally targeting essential cardiogenic genes. In conclusion, we have uncovered a new layer of molecular mechanism by which *HBL1* coordinates with the JARID2/EED-PRC2 complex in the nucleus and microRNA-1 in the cytoplasm to orchestrate the epigenetic state of essential cardiogenic genes and govern cardiogenesis from hPSCs. As lncRNAs could interact with protein factors via specific RNA domains, which relies heavily on 3D RNA structures, it remains unknown whether interspecies conservation of lncRNA should be determined by conserved functional structure(s) rather than solely by sequence (Johnsson et al., 2014). Here, given that the full *HBL1* sequence is not conserved in mouse, our findings at least suggest that lncRNA could play a species-specific role in fine tuning human cardiogenesis.

## MATERIALS AND METHODS

### hPSC lines

hiPSC line S3 (Liu et al., 2017) and H9 ESCs were maintained on mouse embryonic fibroblasts (MEFs) with knockout serum replacement (KSR) medium containing 10 ng/ml FGF2 (KSR medium with FGF2). In a feeder-free culture system, hiPSC line S3 cells and hESC line H9 were maintained on Matrigel-coated plates (BD Biosciences) in mTesR medium. *HBL1*^−/−^ cells were generated in our previous study, in which *HBL1* was knocked out in hiPSCs using CRISPR/Cas-9 technology (Liu et al., 2017).

### Cardiac differentiation

For monolayer CM differentiation, CMs were induced using a previously established protocol (Burridge et al., 2014). Briefly, stem cells were treated with CDM3 medium containing 6 μM CHIR99021 from D0 to D2, 5 μM XAV from D2 to D4, then maintained in CDM3 medium without any chemicals after D4 and fresh medium was changed every 2 days. Beating cells were observed at D8-D13 after differentiation. At D12-D15, cells were collected for experiments. For EB differentiation, stem cells were differentiated towards CMs using a previously established protocol (Lin et al., 2012; Yang et al., 2008). Cardiac differentiation was conducted with EB formation. EBs were treated with StemPro^®^-34 SFM (1×) medium (Thermo Fisher Scientific) with the following conditions: D0-D1 with BMP4 (5 ng/ml); D1- D4 with BMP4 (10 ng/ml), FGF2 (5 ng/ml) and activin A (2 ng/ml); and D4-D20 with or without XAV (5 µM), after D4 the medium was changed every 2 days. Beating cells were observed at D10-D13 after differentiation. On D20, EBs were collected for experiments. All cytokines were from R&D Systems. All chemicals were from Sigma-Aldrich.

### Affinity pulldown of biotinylated RNA followed with mass spectrometry

*HBL1* RNA was transcribed *in vitro* with the T7 promoter using TranscriptAid T7 High Yield Transcription Kit (Thermo Fisher Scientific, K0441) according to the manufacturer's instructions. Then *HBL1* RNA was labeled with desthiobiotin at 3′ using Pierce RNA 3′ Desthiobiotinylation Kit (Thermo Fisher Scientific, 20163). For affinity pulldown, 1×10^7^ hESCs were resuspended in 50 µl RIP Lysis Buffer (with protease inhibitor cocktail and RNase inhibitor) and the whole-cell lysis was extracted using the EZ-Magna RIP™ RNA-Binding Protein Immunoprecipitation Kit (Millipore) according to the manufacturer's instructions. Then 1 µg biotinylated RNA was added into the 50 µl protein lysis and the mixture was incubated at 4°C overnight. Affinity pulldown experiments were performed using Pierce™ Magnetic RNA-Protein Pull-Down Kit (Thermo Fisher Scientific, 20164) according to the manufacturer's instructions. Biotin-labeled anti-sense *HBL1* RNA was used as the negative control. At the end of affinity pulldown, beads were washed with RNase-free H_2_O and submitted to the proteomics core where they were covered in 8 M urea, 50 mM Tris-HCl (pH 8.5), reduced with 5 mM Tris (2-carboxyethyl) phosphine hydrochloride (TCEP) at room temperature for 30 min and alkylated with 10 mM chloroacetamide (CAM) for 30 min in the dark at room temperature. Digestion was carried out using Trypsin/Lys-C Mass spec grade protease mix (Promega, V5072) at a 1:100 protease to substrate ratio overnight at 37°C. The reaction was quenched with 0.5% formic acid before liquid chromatography-mass spectrometry. Samples were analyzed using a 5 cm trap column and 15 cm (2 µm particle size, 50 µm diameter) EasySpray (801A) column on an UltiMate 3000 HPLC and Q-Exactive Plus mass spectrometer (Thermo Fisher Scientific). Solvent B was increased from 5%-28% over 155 min, to 35% over 5 min, to 65% over 10 min and back to 5% over 12 min (Solvent A: 95% water, 5% acetonitrile, 0.1% formic acid; Solvent B: 100% acetonitrile, 0.1% formic acid). A data-dependent top 20 acquisition method was used with mass spectrometry scan range of 350-1600 m/z, resolution of 70,000, AGC target 3e6, maximum IT of 50 ms. MS2 settings of fixed first mass 100 m/z, normalized collision energy of 36, isolation window of 1.5 m/z, resolution of 35,000, target AGC of 1e5, and maximum IT of 250 ms. For data-dependent acquisition a minimum AGC of 2e3 and charge exclusion of 1, and ≥7 was used. A full list of proteins pulled down by biotinylated *HBL1* could be found in Table S1.

### Vector cloning

Two specific shRNA1 and shRNA2 against human EED and shRNA scramble control were cloned into the pLKO.1-TRC vector, separately. To generate the luciferase activity reporter, *JARID2* 3′ UTR containing a putative miR-1 binding site was cloned into the pmiR-GLO vector. To clone a vector with a *JARID2* 3′ UTR with mutated miR-1 binding site, overlap-PCR was performed for site-directed mutagenesis as previously described (Urban et al., 1997).

### Virus package and lentiviral transduction

The lentiviral constructs including pLKO.1-TRC and lentiCRISPRv2-puro vectors were transfected into the HEK293T cells (ATCC) along with lentiviral packaging plasmids including psPAX2 and pMD2.G using the X-tremeGENE 9 transfection reagent (Roche), following the manufacturer's instructions, in Opti-MEM (Life Technologies) and incubated for 3-4 h at 37°C. After incubation for 24-48 h, the viral supernatant was collected and cellular debris was removed by syringe filtering (0.22 μm pore size; Millipore). For lentiviral transduction, lentivirus was added twice, 24 h after cell seeding and again after an additional 24 h. For every infection, S3 hiPSCs or H9 hESCs were infected by viruses overnight. Puromycin (1.0 μg/ml) treatment was used for selection of transduced PSCs after 3 days of virus infection and maintained throughout culture.

### RNA isolation, cDNA synthesis and RT-qPCR

RNA isolation was performed using the RNeasy Mini kit (Qiagen) with additional DNase step following the manufacturer's protocol. cDNA was synthesized using the High-Capacity RNA-to-cDNA™ Kit (Applied Biosystems). RT-qPCR was performed on a 7900HT Fast Real-Time PCR System (Applied Biosystems) with Fast SYBR Green Master Mix (Applied Biosystems). The results were normalized to *GAPDH* gene expression. miRNA cDNA was synthesized using the qScript™ microRNA cDNA Synthesis Kit (Quanta). The results were normalized to *SNORD44* gene expression. RT-qPCR results were presented as mean±s.d. from at least three independent experiments. RT-PCR was performed using Thermo Fisher Scientific DreamTaq Green PCR Master Mix. Primers are presented in Table S5.

### ChIP-qPCR

H9 hESCs were maintained in mTesR medium on a Matrigel-coated P10 plate. ChIP was performed in undifferentiated hESCs using the truChIP™ Chromatin Shearing Kit (Covaris, PN 520154) and EZ-Magna ChIP™ A/G Chromatin Immunoprecipitation Kit (Millipore, 17-10086) according to the manufacturers’ instructions. Briefly, H9 hESCs were fixed with methanol-free formaldehyde provided by the truChIP™ Chromatin Shearing Kit. Sonication of cell lysis was performed by a ME220 Focused-ultrasonicator (Covaris) using truChIP Chromatin Shearing Tissue Kit. ChIP was performed using the EZ Magna ChIP A/G Chromatin Immunoprecipitation Kit. Human EED antibody [1:100,Activemotif: (mAb) RRID: AB_2615071, clone: 41D, Catalog No: 61203], JARID2 antibody [1:100, Cell Signaling Technology: JARID2 (D6M9X) rabbit mAb #13594] and H3K27me3 antibody (1:100, Sigma-Aldrich: 17-622) were used to pull down chromatin. Normal mouse/rabbit IgG or RNA Polymerase II antibodies (provided by Millipore ChIP kit) were used as the negative or positive control, respectively. ChIP-qPCR signals were calculated as fold enrichment of 1% input or non-specific antibody (isotype IgG antibodies) signals with at least three technical triplicates. Each specific antibody ChIP sample was normalized to its isotype IgG antibody-ChIP-signals obtained in the same sample. Error bars represent s.d. (calculated from technical triplicates). Primers for ChIP-qPCR are presented in Table S5.

### ChIP-seq

hESC line H9 was cultured in mTesR medium on a P10 plate. ChIP was performed in undifferentiated hESCs according to the manufacturers’ instructions for the truChIP™ Chromatin Shearing Kit and EZ-Magna ChIP™ A/G Chromatin Immunoprecipitation Kit. Briefly, H9 cells were fixed with methanol-free formaldehyde provided by truChIP™ Chromatin Shearing Kit. Chromatin of cell lysis was sheared using the truChIP™ Chromatin Shearing Kit according to the manufacturer's instructions using a ME220 Focused-ultrasonicator. The sheared chromatin was incubated with anti-EED and anti-H3K27me3 antibodies and purified using an EZ-Magna ChIP™ A/G Chromatin Immunoprecipitation Kit. Chromatin DNA quality was assessed using a 2100 Bioanalyzer (Agilent Technologies) and sequenced at the Center for Medical Genomics at Indiana University School of Medicine, USA.

### ChIRP

For probes, anti-sense oligo DNA probes targeting *HBL1* were designed by using the online probe designer at www.singlemoleculefish.com. Anti-sense oligo DNA probes were synthesized and labeled with biotin by Integrated DNA Technologies. We collected 1.0×10^7^ human S3 iPSC cells cultured in mTesR medium, which were cross-linked and lysed by EZ-Magna ChIRP RNA Interactome Kit (Sigma-Aldrich, 17-10495). The chromatin was isolated by 5′-biotinylated anti-sense oligo DNA probes according to the manufacturer's instructions. Probes sequences are presented in Table S5.

### Flow cytometry

For CM differentiation detection from EBs, flow cytometry was performed according to an established protocol (Yang et al., 2008). Briefly, EBs were harvested and dissociated with Collagenase B for 30 min, followed with 0.25% trypsin-EDTA for 5 min at 37°C. The single cells were fixed in 4% paraformaldehyde (PFA) for 10 min at room temperature, and washed three times with 1× PBS. Cells were incubated in blocking PBS buffer containing 1% bovine serum albumin (BSA) and 0.1% saponin. Then cells were incubated with anti-CTNT antibody (Table S6) diluted with blocking PBS buffer for 1 h at 37°C, followed by secondary antibody diluted with blocking PBS buffer for 1 h at 37°C. CMs generated using monolayer differentiation method were treated with 0.25% trypsin for 10 min then collected and washed with 1× PBS. After washing, cells were fixed with 4% PFA for 10 min at room temperature. Then cells were incubated in blocking 1× PBS buffer containing 1% BSA and 0.1% saponin, followed with secondary antibody diluted with blocking 1× PBS buffer for 1 h at 37°C. Flow cytometry analysis was performed with AccuriC6 flow cytometer (Becton Dickinson). Data were analyzed using FlowJo (Treestar).

### Immunofluorescence staining

Cells were fixed in 4% PFA for 10 min at room temperature and washed in 1× PBS three times. Cells were incubated in primary antibody buffer (containing primary antibody, 1% BSA, 0.1% saponin and 1× PBS) for 1 h at 37°C. After that, cells were washed in 1× PBS three times, then they were stained with secondary antibody buffer (containing secondary antibody, 1% BSA, 0.1% saponin and 1× PBS) for 1 h at 37°C. Finally, cells were washed three times in 1× PBS then stained with DAPI (1:1000) and mounted with Flouromount-G (SouthernBiotech) for imaging. Primary antibodies were: anti-NKX2.5 (Developmental Studies Hybridoma Bank, PCRP-NKX2-5-3B4, RRID: AB_2618896, 1:500) and anti-CTNT (Thermo Fisher Scientific, MS-295-P, RRID: AB_61806, 1:1000). Secondary antibodies were Alexa Fluor 488 polyclonal antibody (Invitrogen, A-11094, RRID: AB_221544, 1:1000) and goat anti-mouse IgG (H+L) cross-adsorbed secondary antibody, Cyanine3 (Invitrogen, A10521, RRID: AB_2534030, 1:1000) (Table S6).

### Dual luciferase assay

The luciferase assay was performed using the luciferase assay system (Promega) according to the manufacturer's instructions. Briefly, HEK293T cells, cultured in 12-well plates, were transfected with luciferase reporter constructs using X-tremeGENE 9 transfection reagent (Roche). Cells were harvested 24 h post-transfection. The firefly and *Renilla* luciferase activity in cell lysates was measured using GLOMAX multi detect system (Promega). We used 100 nM of control or hsa-miR-1 mimic (Invitrogen) and 200 ng of *JARID2* 3′ UTR WT or mutant vector (pmiR-GLO vector) for each well.

### CRISPR/Cas-9-mediated genome editing

The gRNAs were designed using the CRISPR design platform (http://crispr.mit.edu/). Dual gRNAs were used to completely knock out human *JARID2*. gRNAs targeting exon 2 and exon 17 of human *JARID2* were cloned into pENTR-spCas9-EGFP, separately. Two gRNA vectors were co-transfected into the H9 hESC cell line. After 24 h, GFP^+^ H9 cells were sorted by the FACSAria II cell sorter (BD Biosciences) and re-seeded to generate single clones, cultured in mTesR medium with ROCK inhibitor Y-27632. One week later, single H9 clones were picked out and replated into multi-well plates to expand in mTesR medium. Genomic DNA of single clones was extracted using the DNeasy Blood and Tissue kit (Qiagen). Different primer sets (Table S5) were designed to verify whether *JARID2* was knocked out. To disrupt human *EED* transcription, a gRNA targeting the *EED* promoter near the TSS was cloned into the lentiCRISPRv2-puro (Sanjana et al., 2014) vector. A surveyor assay (see below) was used to verify genome editing.

### CRISPR/Cas-9-mediated genome editing on the *EED* promoter

To disrupt human *EED* transcription, a single gRNA specifically targeting *EED* promoter near the TSS was designed using the CRISPR design platform (http://crispr.mit.edu/) and cloned into the lentivirus vector lentiCRISPRv2 (Sanjana et al., 2014) with a puromycin selection marker. For the lentivirus package, the lentiviral lentiCRISPRv2-*EED*-promoter gRNA vector was transfected into the HEK293T cells (ATCC) with lentiviral packaging plasmids including psPAX2 and pMD2.G using the X-tremeGENE 9 transfection reagent (Roche), following the manufacturer's instructions. After incubation for 48 h, viral supernatant was collected and cellular debris was removed by syringe filtering (0.22 μm pore size; Millipore). For lentiviral transduction, lentivirus was added twice, 24 h after cell seeding and again after an additional 24 h. For every infection, H9 hESCs were infected by viruses overnight. Puromycin (1.0 μg/ml) treatment was used for selection of transduced H9 hESCs after 3 days of virus infection and maintained throughout culture. A stable cell line with *EED* knockdown was generated after puromycin selection. A surveyor assay (see below) was used to verify genome editing of the cells after puromycin-consistent selection. For the cardiac differentiation assay, hESCs generated from a single clone were expanded and confirmed by PCR followed with DNA-sequencing. RT-qPCR was performed to evaluate *EED* knockdown efficiency.

### Surveyor assay

Genomic DNA editing was detected using a Surveyor Mutation Detection Kit for Standard Gel Electrophoresis (Integrated DNA Technologies) according to the manufacturer's instructions. Briefly, genomic DNA was purified, followed by PCR, and PCR fragments were extracted. DNA was annealed in 1× prime star buffer (Takara) and the surveyor enzyme was added to digest the annealed DNA fragments, followed with gel electrophoresis to detect the DNA bands after digestion.

### RIP

RIP experiments were conducted using the EZ-Magna RIP™ RNA-Binding Protein Immunoprecipitation Kit (Millipore) according to the manufacturer's instructions. Briefly, 1×10^4^ hESCs were re-suspended in 50 µl RIP Lysis Buffer with protease inhibitor cocktail and RNase inhibitor. For each RIP immunoprecipitation, 5 μg antibody was used and the 1 μg isotype IgG antibody was used as the control. RIP-qPCR results were calculated as fold enrichment versus non-specific antibody (isotype IgG antibody) signal. Error bars represent s.d. (calculated from technical triplicates). Primers are presented in Table S5.

### Co-IP

Co-IP experiments were performed using the Pierce Biotechnology Classic Magnetic IP/Co-IP Kit, according to the manufacturer's instructions. Specific JARID2 antibody [Cell Signaling Technology: JARID2 (D6M9X) rabbit mAb #13594] was used to pull down interacting proteins. Mouse or rabbit IgG was used as control antibody. Protein pulled down by Co-IP was used for western blotting.

### REMSA

The REMSA experiment was performed using the LightShift^®^ Chemiluminescent RNA EMSA Kit (Pierce Biotechnology) according to the manufacturer's instructions. IRE Control RNA from this kit was biotinylated. *HBL1* DNA template was generated by PCR using specific primers containing T7 promoter sequences (Table S5). *HBL1* RNA was transcribed *in vitro* using the DIG Northern Starter Kit (Roche), followed by DIG-labeling. EED recombinant protein was purchased from R&D Systems.

### RNA-seq data analysis

The sequencing reads were mapped to the human genome hg38 using RNA-seq aligner STAR (v2.5) (Dobin et al., 2013) with the following parameter: ‘--outSAMmapqUnique 60’. Uniquely mapped sequencing reads were assigned to hg38 annotation using featureCounts (v1.6.2) (Liao et al., 2014) with the following parameters: ‘-s 2 –p –Q 10’. Genes were filtered out for further analysis if they had read counts >9 in less than two samples. Gene expression profiles for WT and KO were normalized using the trimmed mean of M values (TMM) method and subjected to differential expression analysis using edgeR (v3.24.3) (McCarthy et al., 2012; Robinson et al., 2010). Differentially expressed genes (DEGs) were identified if genes had |log_2_FC|>0.5 and FDR-adjusted *P*-values<0.05.

### ChIP-seq data analysis

ChIP-seq reads were aligned to the human genome (hg38) using Bowtie2 (Langmead and Salzberg, 2012). After duplicated reads were removed by Picard (Broad Institute; version 2.17.8), peak calling of mapped high-quality ChIP-seq reads was performed by MACS2 (Zhang et al., 2008). Peaks were determined after comparison to the input reads with a Benjamini-Hochberg procedure, adjusted *P*-value (*q*-value) <0.01. Peaks locating within the blacklist regions by ENCODE (Amemiya et al., 2019; ENCODE Project Consortium, 2012) were removed. We merged overlapped peaks from multiple samples to form a final set of unique regions across all samples. Binding signals within regions were evaluated for individual samples by reads detected within the region, counted by the FeatureCounts, followed by TMM normalization. Differential analysis was conducted by using edgeR (v3.24.3) to compare the signal differences between WT and *HBL1*^−/−^ samples. The regions with differential EED/H3K27me3 binding signals were identified by *P*<0.05. Heatmaps of ChIP-seq signals in selected regions were plotted using deepTools (Ramírez et al., 2014).

### Functional enrichment analysis

Functional enrichment analysis on DEGs and a set of genes associated with decreased EED/H3K27me3 signals was performed using DAVID (https://david.ncifcrf.gov, v6.8; Dennis et al., 2003). A number of GO functions and KEGG pathways were recognized as significantly over-represented (FDR-adjusted *P*-values<0.05 based on multiple-test correction) in either up- or downregulated DEGs, or a specific gene set. Only cardiac-related GO functions were selected to be presented in this article. The signaling pathway analysis was performed using IPA (Qiagen).

### Quantification and statistical analysis

All data comparisons between groups were analyzed using a two-tailed unpaired Student’s *t*-test (two groups), a one-way ANOVA (multiple groups) or hypergeometric distribution. All data are presented as mean±s.d. from at least three independent experiments. Differences with *P*-values less than 0.05 are considered significant.

## Supplementary Material

Supplementary information
