# Supplementary material for: LncRNA HBL1 is required for genome-wide PRC2 occupancy and function in cardiogenesis from human pluripotent stem cells
Source: Development. 2021 Jun 28;148(13):dev199628. doi: 10.1242/dev.199628 (PMC8276986; doi:10.1242/dev.199628)
Supplement: Supplementary information [file develop-148-199628-s1.pdf]

A

Molecular and Cellular Functions

Name	p-value range	# Molecules
Cell cycle	1.11E-02 - 1.03E-08	47
Cellular Assembly and Organization	1.11E-02 - 4.02E-07	88
Cellular Function and Maintenance	1.11E-02 - 4.02E-07	79
Protein Synthesis	2.38E-03 - 1.60E-08	36
Cellular Compromise	2.50E-03 - 2.18E-08	15

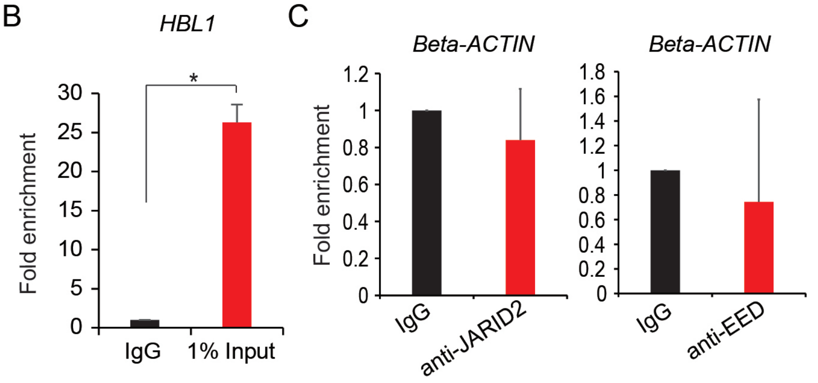

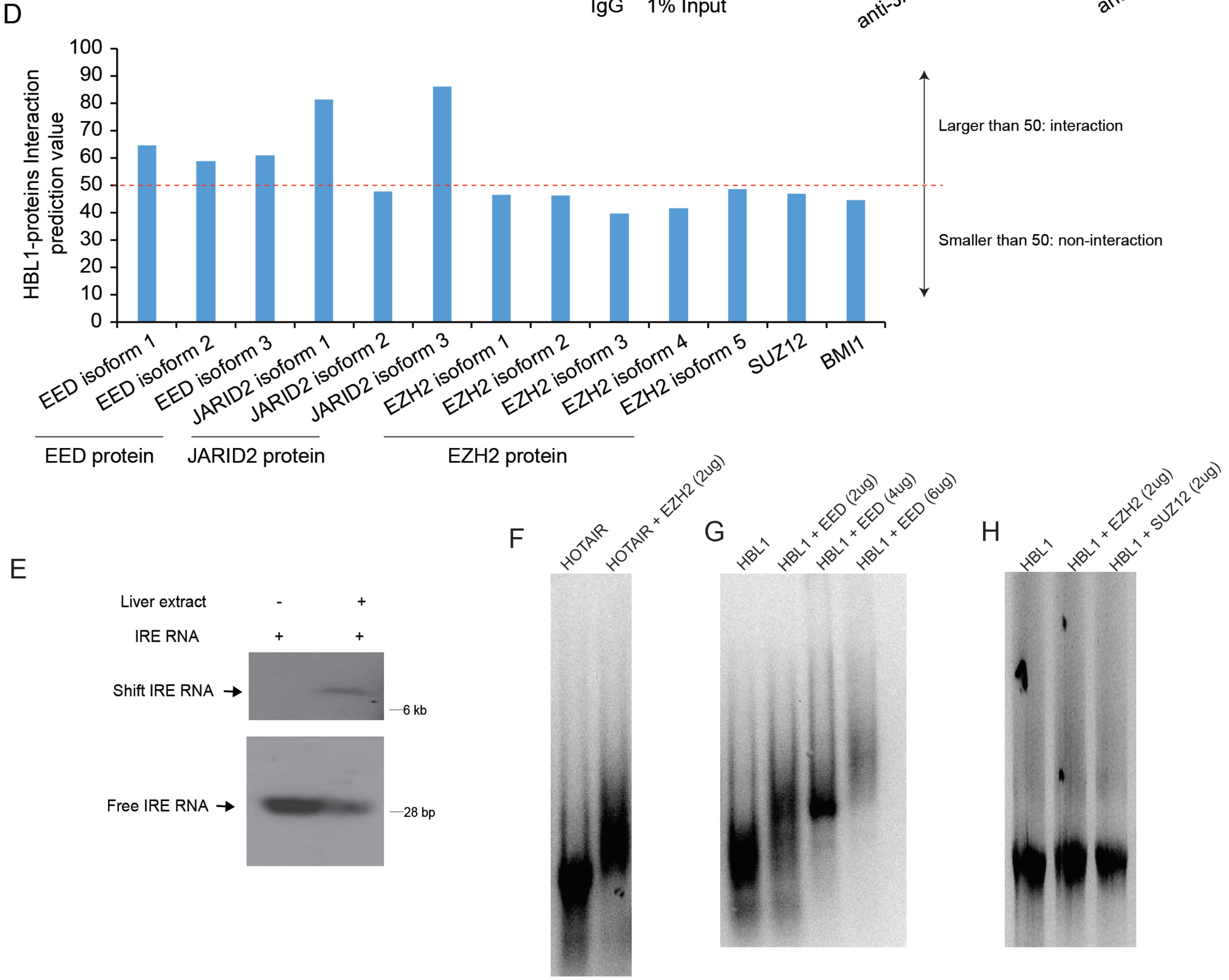

Figure S1. HBL1 interacts with EED and JARID2
(A) Molecular and cellular functions analysis for all protein candidates from MS data.
(B) 1% Input is the RIP control to detect HBL1 expression.
(C) Negative RNA (beta-ACTIN RNA) control detection for RIP-RT-qPCR using JARID2 and EED antibodies. Experiments were performed in triplicate. All bars are shown as mean ± SD. n=3, *p < 0.05 (Student's t-test).
(D) LncRNA-protein interaction prediction analysis shows the interaction values of HBL1 and different epigenetic factors. Prediction was performed by lncPro (http://bioinfo.bjmu.edu.cn/lncpro/#).
(E) RNA electrophoretic mobility shift assay (REMSA) shows that mobility of IRE RNA could be retarded by adding liver extract, which was used as the positive control for REMSA.
(F) HOTAIR lncRNA interacting with EZH2 is used as the positive control for RNA EMSA. HOTAIR amount is 500ng.
(G) REMSA experiment shows the interaction between HBL1 and EED. HBL1 amount is 500ng.
(H) REMSA experiment shows no interaction between HBL1 and EZH2 or SUZ12. HBL1 amount is 500ng.
Relative to Figure 1.

Development • Supplementary information

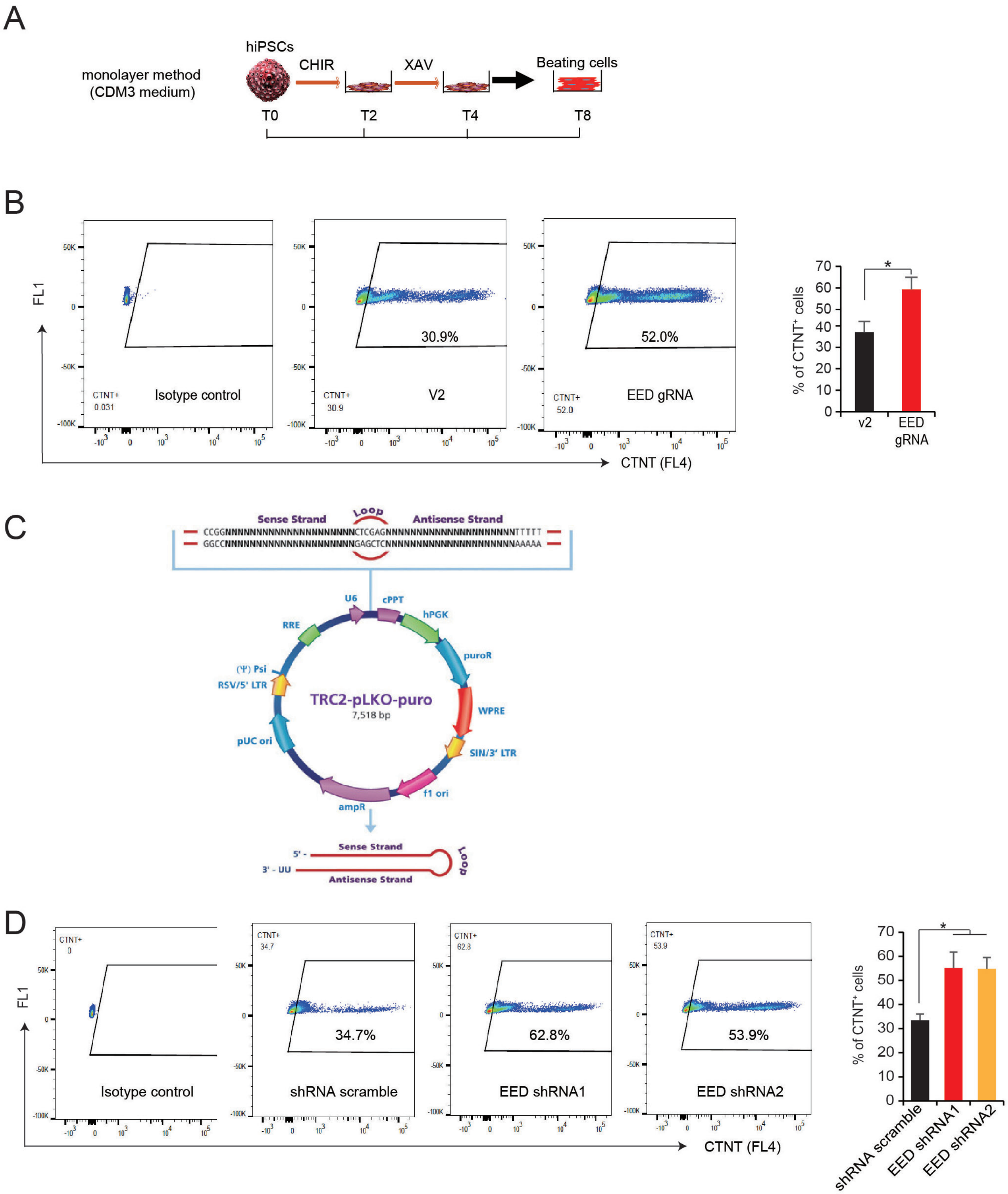

Figure S2. Knockdown of EED promotes human cardiac differentiation

(A) Cardiac differentiation using monolayer differentiation protocol.

(B) Percentage of CTNT^{+} CMs was measured on EEDknockdown S3 hiPSCs by gRNA and CRISPR/Cas9 after cardiac differentiation for 8 days under a monolayer differentiation condition.

(C) PLKO.1-TRC shRNA vector is used to knock down gene expression.

(D) Percentage of CTNT^{+} CMs was detected on EEDknockdown S3 hiPSCs by shRNAs after cardiac differentiation for 8 days under a monolayer differentiation condition.

All bars are shown as mean ± SD. n=3, *$p < 0.05$.

All data comparisons use a one-way ANOVA (multiple groups) or t-test (two groups).

Relative to Figure 2.

Development • Supplementary information

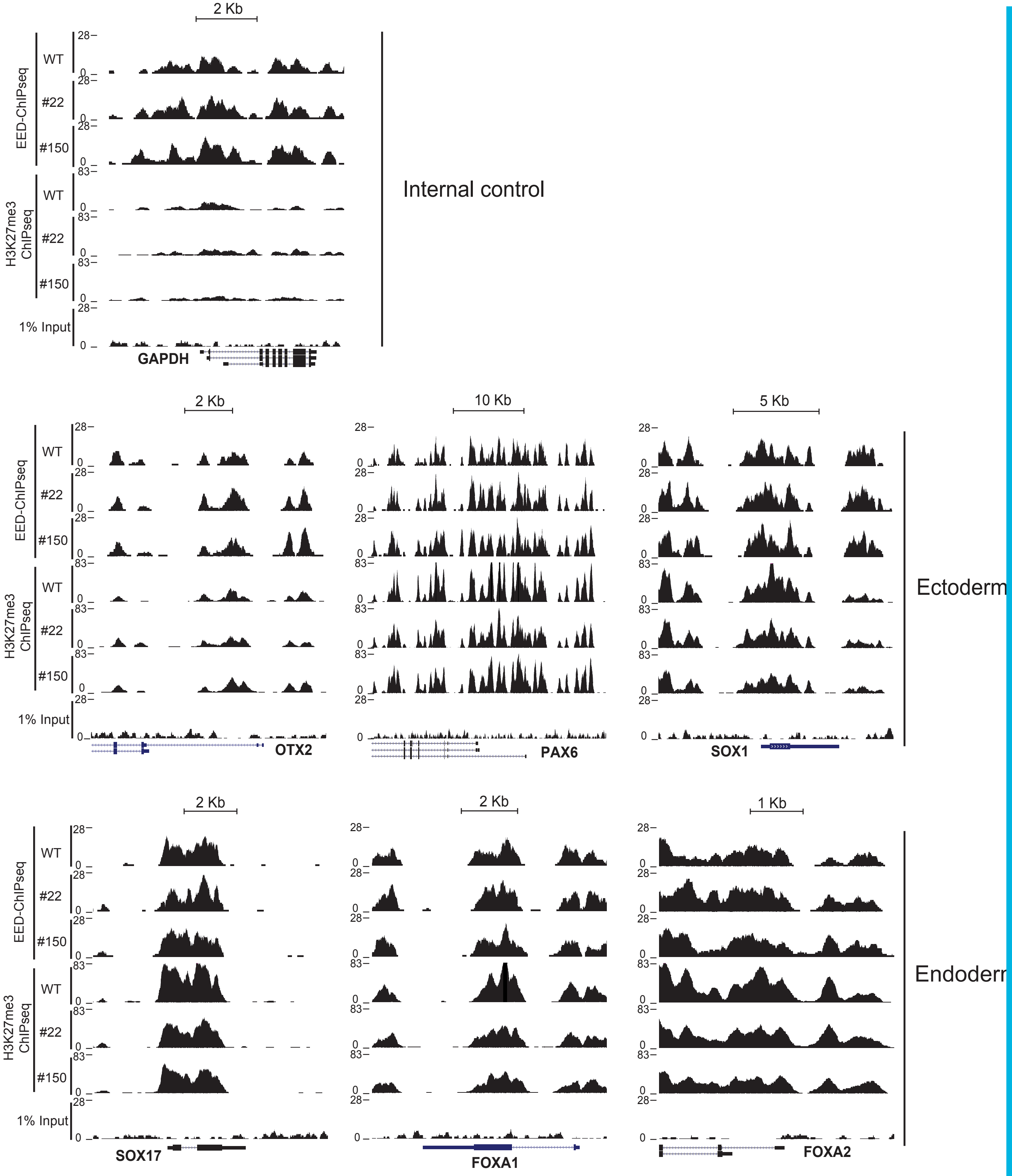

Figure S3. Loss of HBL1 can not affect EED or H3K27me3 occupancies on other lineage markers
Representative genome browser peak tracks of different genes with non-changed EED/H3K27me3 binding (HBL1$^{-/-}$ vs. WT). WT, wild type hiPSCs. #22 and #150 are two HBL1$^{-/-}$ hiPSCs clones. 1% Input is the control.

Relative to Figure 3.

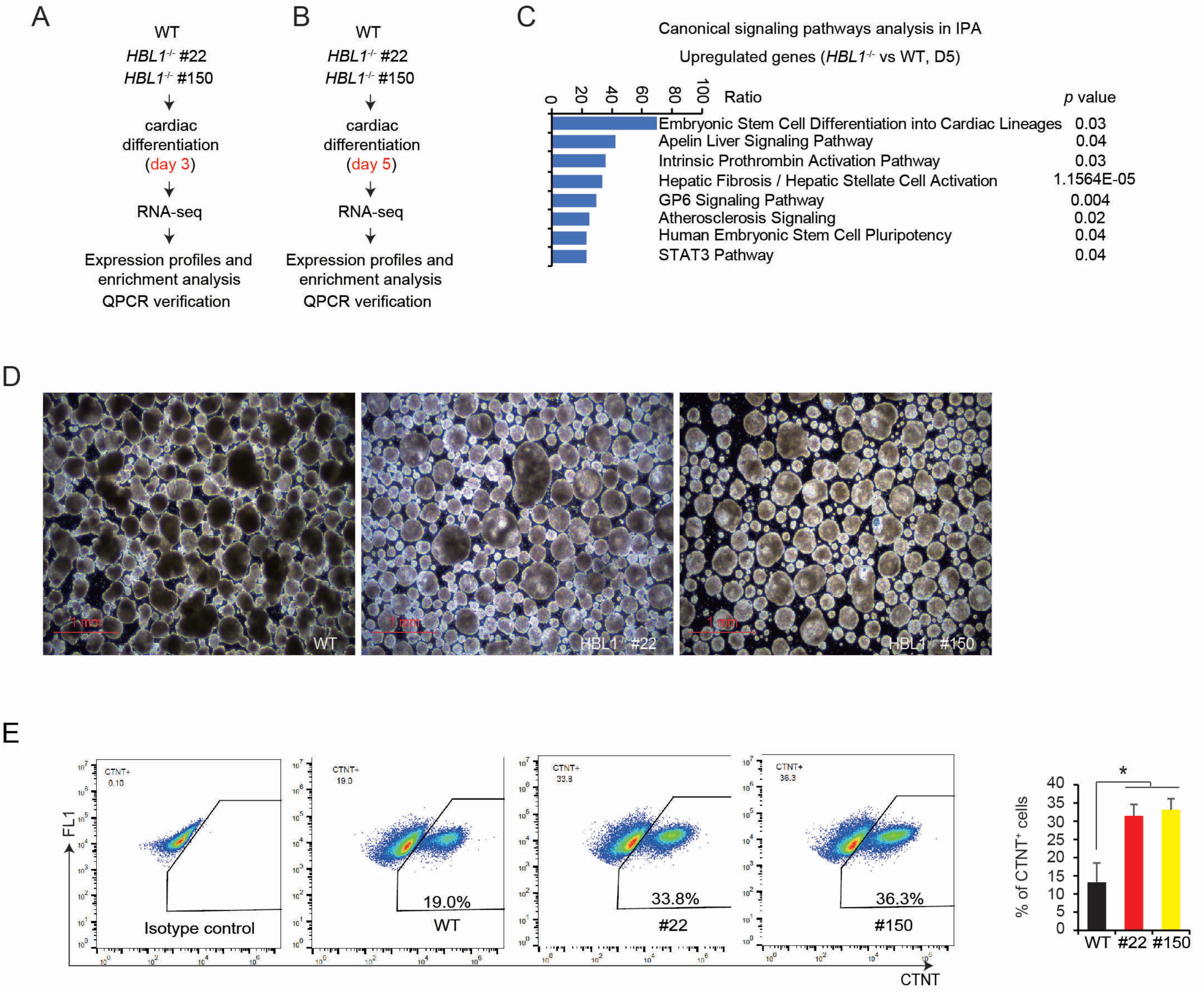

Figure S4. Loss of HBL1 promotes cardiac differentiation from hPSCs

(A-B) RNA-seq was performed on WT and HBL1$^{-/-}$ hiPSCs clones (#22, #150) with 3 days (A) and 5 days (B) cardiac differentiation

(C) Canonical signaling pathways analysis using IPA software.

(D) EBs formation on day 6 differentiation.

(E) After 20 days of cardiac differentiation by forming EBs, percentage of CTNT^{+} CMs was quantified by flow cytometry.

All experiments were performed in triplicate. All bars are shown as mean ± SD. n=3, *$p < 0.05$ (one-way ANOVA).

Relative to Figure 4.

Development • Supplementary information

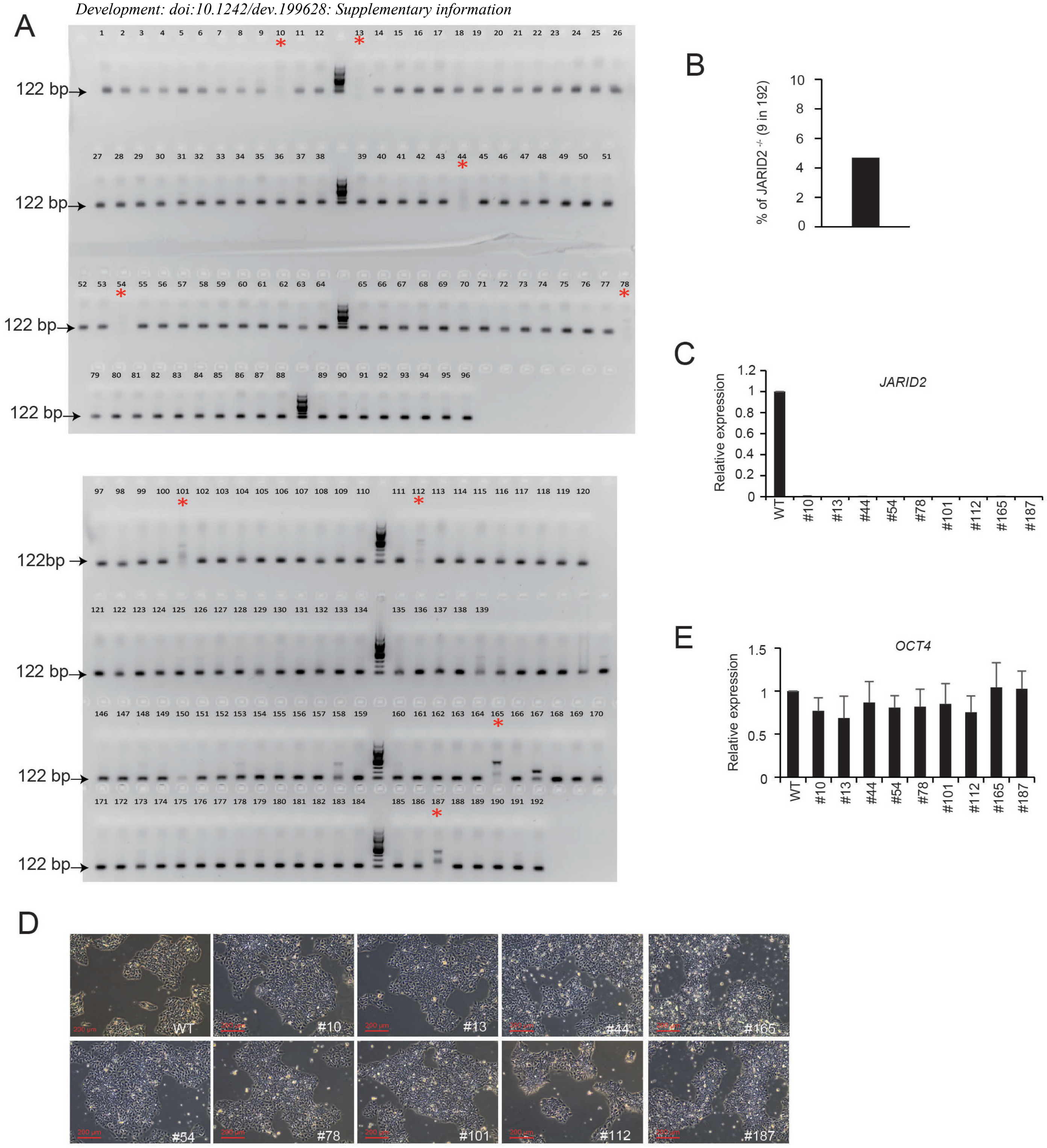

Figure S5. Knockout of JARID2 in H9 hESCs
(A) PCR to screen JARID2$^{-/-}$ H9 hESC clones using specific primers.
(B) JARID2 knockout efficiency analysis in all H9 hESC clones.
(C) MRNA expression of JARID2 in WT H9 and JARID2$^{-/-}$ H9 clones.
(D) WT and JARID2$^{-/-}$ hESC clones cultured in mTesR1 medium.
(E) MRNA expression of OCT4 in WT and JARID2$^{-/-}$ hESC clones.
Experiments were performed in triplicate. All bars are shown as mean ± SD. n=3, *$p < 0.05$ (one-way ANOVA).

Relative to Figure 5.

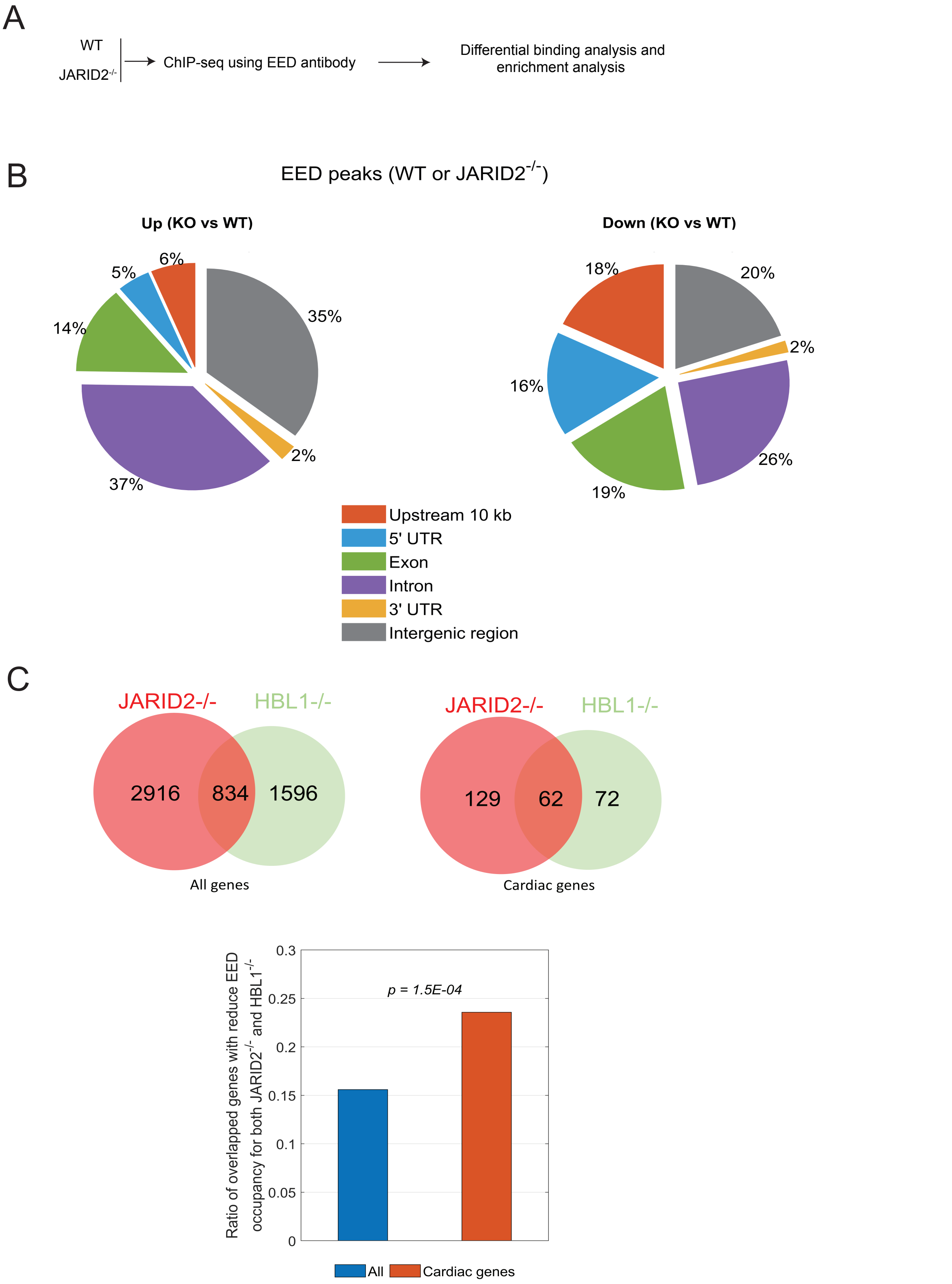

Figure S6. Genome-wide EED occupancy is regulated by JARID2.
(A) EED ChIP-seq was performed on WT and JARID2$^{-/-}$ hESCs.
(B) EED binding peaks with increased (left) and decreased (right) binding signals (WT vs. JARID2$^{-/-}$).
(C) Overlapped genes analysis in two EED ChIP-seq datasets in JARID2$^{-/-}$ and HBL1$^{-/-}$ hPSCs.
In the top, left one shows the all overlapped genes, right one shows the overlapped cardiac genes.
In the bottom, blue bar shows the ratio of all overlapped genes with reduced EED occupancies, and the red bar shows the ratio of only overlapped cardiac genes with reduced EED occupancies.

Relative to Figure 6.

Development • Supplementary information

A

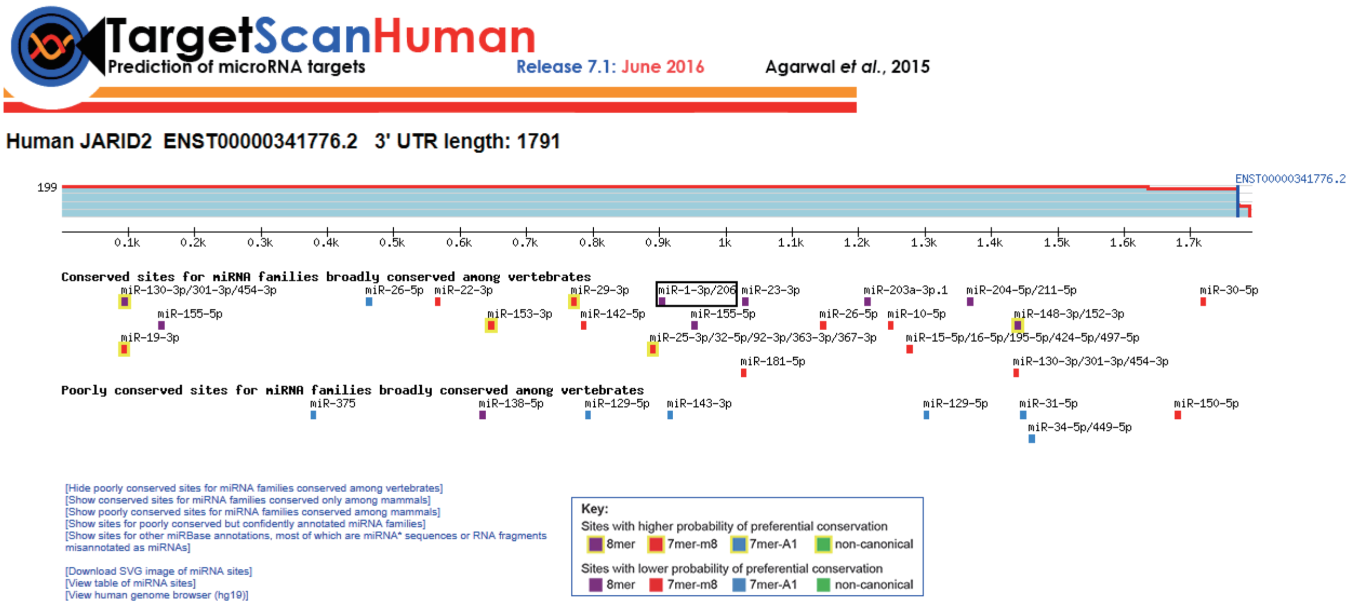

B

Conserved

	Predicted consequential pairing of target region (top) and miRNA (bottom)	Site type	Context++ score	Context++ score percentile	Weighted context++ score	Conserved branch length	P_{CT}
Position 900-907 of JARID2 3' UTR<br>hsa-miR-613	5' ...CGUGCAAUCUUUCUAACAUUCCA...<br>||||||| 3' CCGUUUCUUCCUUGUAAGGA	8mer	-0.28	95	-0.28	4.121	0.75
Position 900-907 of JARID2 3' UTR<br>hsa-miR-1-3p	5' ...CGUGCAAUCUUUCUAACAUUCCA...<br>||| ||||||| 3' UAUGUAUGAAGAAAUGUAAGGU	8mer	-0.27	94	-0.27	4.121	0.75
Position 900-907 of JARID2 3' UTR<br>hsa-miR-206	5' ...CGUGCAAUCUUUCUAACAUUCCA...<br>||| ||||||| 3' GGUGUGUGAAGGAAUGUAAGGU	8mer	-0.27	94	-0.27	4.121	0.75

C

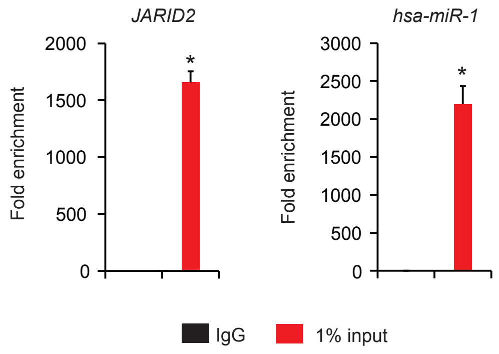

Figure S7. JARID2 is a target of microRNA-1.
(A, B) JARID2 is a highly putative and conserved target of hsa-miR-1.
Putative hsa-miR-1 targets are predicted by using TargetScan software (http://www.targetscan.org/vert_50/).
(C) 1% of Input control for RIP-RT-qPCR to detect JARID2 mRNA and hsa-miR-1 expression in miR-1 OE hiPSCs using AGO2 antibody. Experiments were performed in triplicate. All bars are shown as mean ± SD. n=3, *$p < 0.05$ (Student's t-test).

Relative to Figure 7.

Development • Supplementary information

Supplementary Materials and Methods

Human JARID2 last exon (from NCBI database, including 3'UTR sequence, 3'UTR has 1791 bases. Sequences labeled in box are putative miR-1-3p targeting site.)

GAACAGATTATCAGTCTGGTCAATCAGATCTGCGGCAAAGTGTCTGGTAAAAACGGCAGCATTG
AGAACTGTCTCAGTAAACCCACACCAAAAAGAGGTCCCCGCAAGAGAGCGACAGTGGACGTGCCC
CCCTCCCGTCTGTCAGCCTCCAGTTCATCCAAAAGTGCTTCGAGCTCATCATGAAGATGCCAACG
CCCGTGGTCGATTTATATATATTTTTTTGTAATTATTATATTCTAGTTTGGAGTACTTGCTGTAG
GATTCAAGCTGTCTTTGCACTAGCTCTAAAGAAGATTTTCTTCTGGTTTTAGAGAACTAATTTTG
TTTTAGCATTAAACTGTTGAACTTTTTTTTGTACTTAGAAAACCTAGATACTGCAGTCAGATTTT
GGAAACTGCCGTATAGTCACTGTTTTAAAAACCCCGGAGGGGCTGTATTAATTTGTATTGCCCCA
TGGCTGACAAAAGCCTTTTTTTTTGGTTTTGATTTTTTTTTTTTTGTAACTGTTGGGGGGAAAA
AGGCTTTTTAACCCATTTTTGAAGAGGGTGAAGTTTGGAGAACAAATTTAAAAACCATCAGTCA
TGTGAGCAGATTTTTTAGAAGGGATAGGAGACACACGCGCACACACACACACACACGAAACTTG
AAATGGCTTTGCTTTGGCTGTCGTCTTCTGCCGTGTGCCAGATGAGCTTGTGATCTGGGAAGCCG
GGGCACCCCCGTTTTGTTTCTCTGGGCGGTTGTGGCAGCTGAAGGCGGACGTTGTTTCCTAACCA
TAGGTGGAACGAGGAGACGGGAGCGAGTGGGCTCTCCACCAGCACATCACTATGCATCTGTTCCA
GGAAAGAAGAAAAGCGAGCGAGGAAGACGGAAAAGACTGCCTGCCTTGGAGGGGTCACATGAGG
GAGACCTGTGCCTGATTTCATTAGGAAATCCATTCTGTTATTTTTTGGTGCTGTTGGCTACTTTA
TCAAAAAACCCTTCAATAGCATCCTTAAGATTTAAAAAAAAAAAAAAAAAAAAAGGAAAAAAAA
GTGATGGAAGCCGTAAGTGCTTCTTTGTCATCGACGTGCAATCTTTCTAACATTCCATCTCCATC
TCACCGCTTCTTGTTTGACACCTTCACAAGTCAGCATTAATCTTTCTTTTAAAACTTGTTTCATT
TATGATCATGTAGAGAGCCACTAGGAGGCCTGCAGTTATTTTTGAATGTGAAAATGCATTTGCG
TTCATCTTGTCTATTTTTTCTCTTCATGTTGTAACAAAAAGGAAAAAAGAAAAAAAAATCCCAT
CCCTTTTGTACATATGCCTGTAAATTGTTTTAAATACTTGAGCCTTTTTCTCGGTGGGGGGTGGG
GAGGGGGGTGAGAAGACAAGATGAAGAAAAGCCTTACATTTCAGTTTCTTCATCGGTTGGATTG
GATGCTTACAGGGTTTTTCTTGTAACATTTATAAGTGCTGCTTACATCACTGAACAACAACAAA
AAAATAATAATGGAGTAGCTGTTGCCCTTCTCCGGTTGTGTGTACAGTATGTGTGGAATAAAAA
AGGGAAACTGTTTTCACAAGCTGTTCTTTGTTTCATAATTGGATTCATCAATCCCGTAGCTACCC
ATATTGCACTGAGCTTGCCAGTGGTGACTGCCAGGAACGTCCTATGATCCACTTTGTTGGTTGTT
GTTGCAGAAGACTGAACTGTTTTGGAATATTTAACAATTACAGAAACAGTCAAGTGTTTTCCAA
TGTGGTTGTCCGGTTTCTATGGCCTTGCTGTGTACTTTCCCTCTTTTTGACAGTAAACTTCTGCC
TATGGCTTACAGTTTGACATTTAATTTATTAGCGCTGCTCTGCACCCCTCCCTTGGGAGGGAGAC
TTCATGTGGTTTATTGCGAGTTTTTTGTTTACTTTTCAGGTTTGTACTACAAGGTTTAATAATA
AAAACAAAGTTTTTTGGACATTTGTCTGTCTTGTGGAA

Development • Supplementary information

Prediction and the conservation analysis were done by Targetscan software:

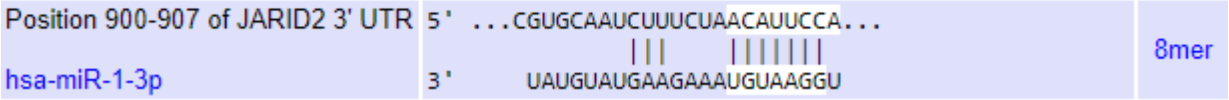

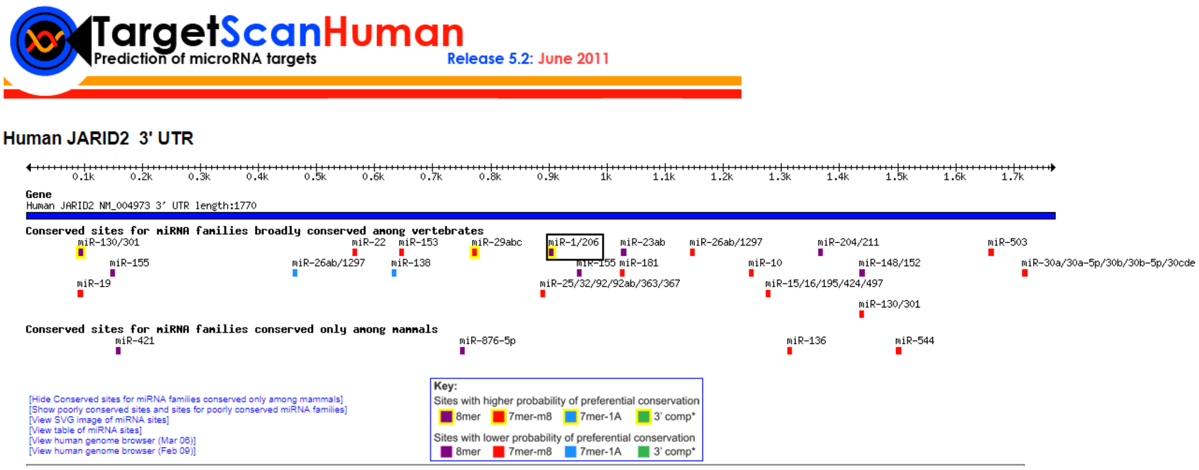

Table S1. Proteins pulled down by biotin-HBL1

Click here to download Table S1

Table S2. EED H3K27me3 ChIP-seq genes_decreased EED/H3K27me3 upstream

Click here to download Table S2

Development • Supplementary information

Table S3. RNA-seq fold change - HBL1-KO vs WT

Click here to download Table S3

Table S4. EED ChIP-seq fold change - JARID2-KO vs WT

Click here to download Table S4

Table S5. Oligonucleotides

Click here to download Table S5

Table S6. Key Resources

REAGENT or RESOURCE	SOURCE	IDENTIFIER
**Antibodies**		
Rabbit IgG	Millipore	MAGNARIP01, RRID:AB_10681285
Mouse IgG	Millipore	MAGNARIP01, RRID:AB_2617156
Mouse IgG Isotype Control	R&D system	MAB002, RRID:AB_357344
Cardiac Troponin T	Thermo Fisher	MS-295-P, RRID:AB_61806
APC goat anti-mouse IgG	BD	550826, RRID:AB_398465
APC-mouse anti-human CD117	R&D Systems	FAB332A-025, RRID:AB_2131315

Development • Supplementary information

NKX2.5	DSHB	PCRP-NKX2-5-3B4, RRID:AB_2618896
ISL1	DSHB	40.2D6 RRID: AB_528315
JARID2	Cell Signaling Technology	13594S RRID: AB_2798269
EED	Millipore	17-663 RRID: AB_10615638
EED	Activemotif	RRID:AB_2615071, Catalog No: 61203
PE-mouse anti-human KDR	R&D system	FAB357P RRID:AB_357165
DDX21	Invitrogen	PA5-30304 RRID:AB_2547778
KMT3A	abcam	Ab200912
ZNF140	DSHB	PCRP-ZNF140-1.2D1) RRID:AB_2619322
HDAC9	abcam	Ab59718 RRID:AB_941883
H3K27me3	Millipore	07-449 RRID:AB_310624
PE Goat anti-mouse	Jackson ImmunoResearch	115-116-146 RRID:AB_2338629
**Chemicals, Peptides, and Recombinant Proteins**		
RhBMP4	R&D system	314-BP
RhFGF2	R&D system	233-FB
RActivin A	R&D system	338-AC
XAV 939	R&D system	3748
DreamTaq Green PCR Master Mix (2X)	Thermo Scientific	K1081
Recombinant Human EED Protein	Novus Biologicals	NBP2-23020
Recombinant Human EZH2 Protein	Novus Biologicals	H00002146-P01-10ug
Recombinant Human SUZ12 Protein	Novus Biologicals	H00023512-Q01-10ug
CHIR99021	R&D system	4423
SYBR Green Master Mix	Applied Biosystems	4385612
**Critical Commercial Assays**		

Development • Supplementary information

truChIP™ Chromatin Shearing Kit	Covaris	PN 520154
Magna ChIP™ A/G Chromatin Immunoprecipitation kit	Millipore	17-10085
EZ-Magna RIP™ RNA-Binding Protein Immunoprecipitation Kit	Millipore	17-701
Surveyor® Mutation Detection Kit for Standard Gel Electrophoresis	Integrated DNA Technologies, Inc.	706021
Dual luciferase assay system	Promega	E2920
High-Capacity RNA-to-cDNA™ Kit	Applied Biosystems	4387406
miRNeasy mini kit	Qiagen	217004
qScript™ microRNA cDNA Synthesis Kit	Quantabio	95107
Classic Magnetic IP/Co-IP Kit	Pierce	88804
DIG Northern Starter Kit	Roche	12 039 672 910
LightShift® Chemiluminescent RNA EMSA Kit	Pierce	20158
**Experimental Models: Cell Lines**		
293T cells	ATCC	CRL-3216
Human iPSC line S3	Carvajal-Vergara et al., 2010	N/A
HBL1 knockout human S3 iPSCs clone 22<br>HBL1 knockout human S3 iPSCs clone 150	This paper	N/A
JARID2 knockout human H9 ESCs clone #10, #13, #44, #54, #78, #101, #112	This paper	N/A
Human S3 iPSCs with expression of EED shRNA1 and 2<br>Human S3 iPSCs with expression of scramble shRNA	This paper	N/A
Human H9 ESCs with expression of EED shRNA 1 and 2<br>Human H9 ESCs with expression of scramble shRNA	This paper	N/A
Human S3 iPSCs with expression of gRNA targeting EED promoter<br>Human S3 iPSCs with expression of gRNA control vector v2	This paper	N/A
Human H9 ESCs with expression of gRNA targeting EED promoter<br>Human H9 ESCs with expression of gRNA control vector v2	This paper	N/A
**Recombinant DNA**		
pmiR-GLO vector	Promega	E1330
pHAGE-puro-inducible vector	From Dr. Gang Hu lab (NIH)	N/A

Development • Supplementary information

pENTR-spCAS9-T2A-EGFP vector	Yi Sheng	N/A
psPAX2	From Dr. Gang Hu lab (NIH)	N/A
pMD2.G	From Dr. Gang Hu lab (NIH)	N/A
pLKO.1-TRC-puro vector	addgene	N/A
lentiCRISPRv2-puro vector	Addgene (Sanjana et al., 2014)	For miR-1 binding mutation
**Software and Algorithms**		
Image J	National Institutes of Health	https://imagej.nih.gov/ij/
FlowJo (Treestar)	FlowJo, LLC	https://www.flowjo.com/about/company

Development • Supplementary information
